# The contribution of the ^240^Ala:Glu:Glu:Thr^243^ sequence in the DE-loop of D2 to the acceptor side of Photosystem II

**DOI:** 10.1007/s11120-026-01222-4

**Published:** 2026-06-16

**Authors:** Ei Phyo Khaing, Tina C. Summerfield, Jack A. Forsman, Imre Vass, Priyanka Pradeep Patil, Julian J. Eaton-Rye

**Affiliations:** 1 Department of Biochemistry, University of Otago, Dunedin, 9054 New Zealand; 2 Department of Botany, University of Otago, Dunedin, 9016 New Zealand; 3 Institute of Plant Biology, Biological Research Center, HUN-REN, Szeged, Hungary; 4 Doctoral School of Biology, Faculty of Science and Informatics, University of Szeged, Szeged, Hungary

**Keywords:** Bicarbonate, Cyanobacteria, D2, Photosynthesis, Plastoquinone, Q_A_, Q_B_

## Abstract

The D2 protein of Photosystem II has five transmembrane helices (A-E). An extended loop, connecting helices D and E, contributes to the binding environments of the primary quinone electron acceptor Q_A_, and that of the bicarbonate bound to the non-heme iron between Q_A_ and the secondary quinone electron acceptor Q_B_. The residues from Ala240 to Thr243 are within a conserved sequence (^240^AEETYSMVTAN^250^) that contributes to the stabilization of both Q_A_ and bicarbonate. We have created the A240D, E241A, E242A, E242D and T243A mutants to study the role of these residues. The mutations in the A240D and E241A strains had little impact on PS II performance, except addition of formate altered chlorophyll *a* fluorescence decay in the E241A mutant following a single-turnover actinic flash. Measurements of variable chlorophyll *a* fluorescence and thermoluminescence showed the E242A and E242D mutants had impaired acceptor side electron transport consistent with a reduced redox gap between Q_A_ and Q_B_. In addition, the T243A mutant exhibited a heightened susceptibility to photodamage. These data show that mutations introduced between ^241^Glu–Thr^243^ of D2 impair PS II activity but are less detrimental than mutations between the corresponding ^243^Glu–Thr^245^ residues of D1 in the vicinity of the Q_B_-binding site.

**Supplementary Information:**

The online version contains supplementary material available at 10.1007/s11120-026-01222-4.

## Introduction

Photosystem II (PS II) is the thylakoid membrane protein complex that catalyzes the light-driven oxidation of water and reduction of plastoquinone (Shevela et al. 2023; Imaizumi and Ifuku 2025). The water-splitting reaction by PS II is conserved in cyanobacteria, algae and plants, and the cyanobacterium *Synechocystis* sp. PCC 6803 (hereafter *Synechocystis* 6803), employed in this study, has been widely adopted as a model organism to study PS II function (Shen et al. 2021; Gisriel et al. 2022b). In cyanobacteria, the mature PS II complex is found as a dimer of ~ 700 kDa with each monomer containing ~ 20 subunits (Umena et al. 2011). The D2 protein, together with D1, form the central core of the PS II reaction center and these are flanked by the chlorophyll *a*-binding subunits CP47 and CP43 that form a proximal antenna. An additional 13 low-molecular-weight membrane-spanning subunits surround these four core subunits, and three or four extrinsic lumenal subunits are also present (Shen 2015; Gisriel et al. 2022b).

Biogenesis of the photosystem begins with the formation of the reaction center complex, assembled from preformed D1 and D2 modules, that combines with a CP47-containing module. The resulting RC47 complex is then joined by a CP43 module followed by the extrinsic subunits to form an active complex (Komenda et al. 2024). Finally, PS II dimerizes and associates with the phycobilisome antenna system (Domínguez-Martin et al. 2022). In addition, PS II possesses the capacity for self repair in response to oxidative damage induced by stress and side reactions arising from the water-splitting chemisty (Nishiyama et al. 2011; Johnson and Pakrasi 2022).

Light energy from the phycobilisome is passed through the proximal antenna to the chlorophyll molecules that make up the P680 reaction center pigments (Müh and Zouni 2020). Upon absorption of a photon, P680* transfers an electron to the primary plastoquinone electron acceptor Q_A_ via a pheophytin (Cardona et al. 2012). The electron is then transferred to the secondary plastoquinone acceptor Q_B_, and following a second turnover, Q_B_H_2_ is formed and exchanged with an oxidized PQ from a pool of plastoquinone in the thylakoid membrane (Van Eerden et al. 2017). The P680^+^ formed during these steps is reduced by electrons from the Mn_4_CaO_5_ oxygen-evolving complex (OEC) which cycles through 5 (S_0_ to S_4_) oxidation states (Kern et al. 2018).

**Fig. 1:**
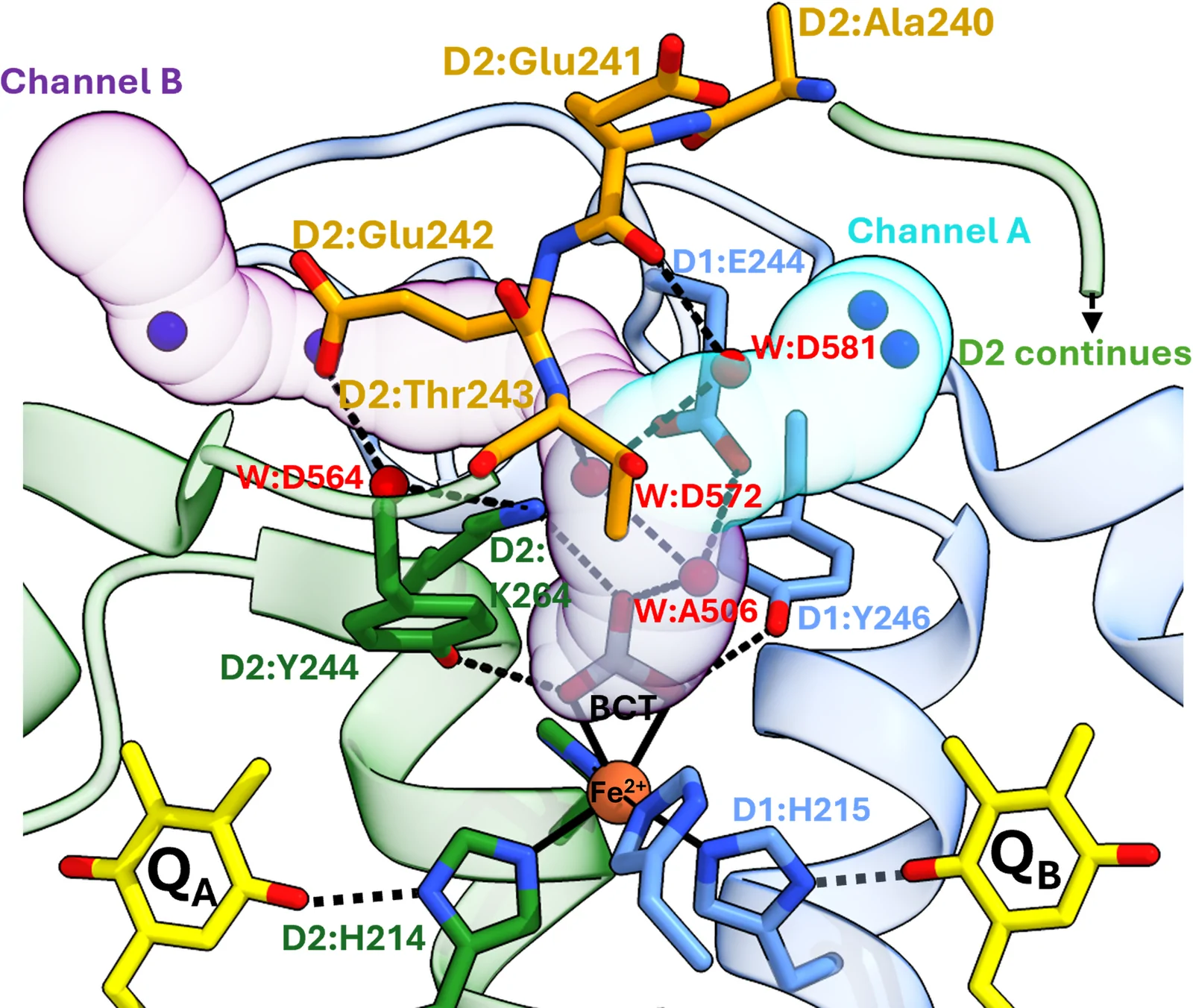
Location of the D2-Ala240 to D2-Thr243 residues in Photosystem II. D1 residues are in blue and D2 residues are in green except for the D2-A240, D2-E241, D2-E242 and D2-T243 residues that are indicated in orange. BCT represents bicarbonate, Fe^2+^ is the non-heme iron (orange sphere). The water molecules indicated in red (W:A506, W:D564, W:D572 and W:D581) are located within 6.3 Å from the bicarbonate ion. The Q_A_ and Q_B_ quinones are shown in yellow. Oxygen atoms are shown in red and nitrogen atoms are shown in blue. Dashed black lines indicate putative hydrogen bonds and represent distances of 3.6 Å or less. Coordination bonds are shown as black lines. Two channels (Channel A and Channel B) from the non-heme iron to the cytosolic surface are shown and were generated using the program CAVER (Jurcik et al. 2018). The W:A506, W:D572 and W:D581 waters are located inside Channel A. Additional waters located in Channels A and B are shown as blue spheres. The figure was generated using PyMOL (Molecular Graphics System, Schrödinger, LLC) and PDB: 7N8O

Efficient electron transfer between Q_A_ and Q_B_ is facilitated by a non-heme iron (NHI) located between the two quinones (Tamura et al. 2021; Sugo et al. 2022b). Axial ligation of the NHI is provided by His215 and His272 of D1 and by His214 and His268 from D2. (Fig. 1). The coordination of the NHI is completed by bidentate ligation via a bound bicarbonate ion (Umena et al. 2011; Shevela et al. 2012). The D1 and D2 proteins possess five transmembrane helices (A–E) with a hydrophilic loop connecting helices D and E on the cytosolic (or stromal) side of the membrane. A conserved motif between ^242^EEETYNIVAAH^252^ in D1 and ^240^AEETYSMVTAN^250^ in D2 surrounds the NHI environment with D1-Tyr246 and D2-Tyr244 providing hydrogen bonds with bicarbonate (Hienerwadel and Berthomieu 1995; Takahashi et al. 2009; Umena et al. 2011). Mutations targetting D1-Tyr264 and D2-Tyr244 severly impaired electron transfer between Q_A_ and Q_B_ and in a D2-Y244A mutant in *Synechocystis* 6803, PS II assembly was disrupted (Forsman et al. 2019a; Khaing et al. 2022; Nihara et al. 2025).

The D1 residues D1-His215, D1-Tyr246 and D1-His252 also play prominent roles in the protonation steps required for the formation of Q_B_H_2_ (Saito et al. 2013; Sugo et al. 2022a). Likwise the D2 residues D2-Met246, D2-Asn250 and D2-Trp253 contribute to the binding of Q_A_ (Vermaas et al. 1990; Zhong et al. 2025). During the assembly of the quinone-iron acceptor complex, the position of the DE-loops of both D1 and D2 is regulated by the Psb28 assembly factor which is released prior to the biniding of both bicarbonate and Q_B_ (Xiao et al. 2021; Zabret et al. 2021; Brown et al. 2024). Given the varied roles played by the D1 and D2 residues in or near the bicarbonate-binding motif, we have investigated the contribution of the conserved D2-Ala240 to D2-Thr243 region to the stability and activity of PS II.

## Materials and methods

### Generation of site-directed mutations and growth of *Synechocystis* 6803 strains

The wild type for this study was the glucose-tolerant *Synechocystis* 6803 GT-O1 strain (Williams 1988; Morris et al. 2014). The mutants: A240D, E241A, E242A, E242D and T243A, were constructed using the Quick change II site-directed mutagenesis kit (Agilent, Santa Clara, CA, U.S.A.) using the primers listed in Table S1. The mutations were introduced into a plasmid that contained the *psbDI:psbC* operon. The plasmid corresponding to each mutant was then introduced into a strain of *Synechocystis* 6803 in which the *psbDI:psbC* operon and the *psbDII* gene had been deleted (Khaing et al. 2020). In the deletion mutant, the *psbDII* gene was replaced by a kanamycin-resistance cassette and the *psbDI:psbC* operon replaced by a chloramphenicol-resistance cassette. Selection for the introduced *psbDI:psbC* operon containing the individual mutations was achieved using a spectinomycin-resistance cassette (Khaing et al. 2020). Colony PCR and Sanger sequencing were used to confirm the presence and segregation of the introduced mutations. This method for constructing mutants was also used to produce a control strain with an unmodified *psbDI:psbC* operon.

Strains were maintained on BG-11 media plates containing 5 mM glucose, 20 µM atrazine, 10 mM TES-NaOH (pH 8.2), 0.3% sodium thiosulfate and appropriate antibiotics with constant light at ~ 30 µmol photons m^− 2^ s^− 1^ (Eaton-Rye 2011). Kanamycin and spectinomycin were used at a final concentration 25 µg mL^− 1^. When present, chloramphenicol was added at 15 µg mL^− 1^. Photoautotrophic growth measurements and preparation of mixotrophic BG-11 liquid cultures were performed as described in Eaton-Rye (2011).

### Measurement of oxygen evolution and photodamage assays

Oxygen evolution was measured with a Clark-type oxygen electrode (Hansatech, King’s Lynn, U.K.) at 30 °C with samples at 10 µg mL^–1^ chlorophyll *a*. For PS II measurements, either 200 µM 2,6-dichloro-1,4-benzoquinone (DCBQ) or 200 µM 2,5-dimethyl-1,4-benzoquinone (DMBQ) was added, both in the presence of 1 mM potassium ferricyanide (K_3_Fe(CN)_6_). For whole-chain oxygen evolution measurements, 15 mM sodium bicarbonate was used. For photodamage and recovery assays, samples at 10 µg mL^–1^ chlorophyll *a* were exposed to white light at ~ 2000 µmol photons m^–2^ s^–1^ using a Kodak Ektalite 1000 slide projector for 45 min followed by white light at ~ 30 µmol photons m^–2^ s^–1^ for 135 min, provided by metal halide bulbs as in Luo and Eaton-Rye (2008). In these assays, oxygen evolution was measured at 15 min intervals using DMBQ with K_3_Fe(CN)_6_ or 15 mM sodium bicarbonate. Photodamage and recovery assays were also performed following the addition of 500 µg mL^− 1^ of lincomycin after the intial T0 measurement, directly before high-light exposure or after 45 min of high-light exposure.

### Measurement of variable chlorophyll *a* fluorescence

An FL-3500 fluorometer (Photon Systems Instruments, Brno, Czech Republic) was used to measure variable chlorophyll *a* fluorescence induction and decay kinetics. To measure fluorescence induction, samples at 5 µg mL^–1^ chlorophyll *a* were dark adapted for 5 min and then measured using a blue actinic light (455 nm) and blue measuring flashes (455 nm) in the presence or absence of 3-(3,4-dichlorophenyl)-1,1-dimethyl urea (DCMU) at 40 µM as described in Jackson et al. (2014).

For the fluorescence decay experiments, fluorescence was measured following single-turnover actinic flashes (455 nm) spaced at 200 ms (5 Hz) in the presence and absence of 40 µM DCMU after being dark adapted for 5 min (Forsman and Eaton-Rye 2021). The fluorescence decays were probed with blue measuring flashes (455 nm). Kinetic analyses were performed according to Vass et al. (1999).

### Low-temperature (77 K) fluorescence emission spectra

A modified MPF-3 L fluorescence spectrometer (Perkin-Elmer, Waltham, MA, U.S.A.) equipped with a custom-made Dewar was used to measure the fluorescence emission at 77 K. Samples at 2.5 µg mL^–1^ chlorophyll *a* were frozen and data collected following excitation at either 440 or 580 nm (Jackson et al. 2014). Spectra were normalized to the emission maximum of Photosystem I (PS I) at 725 nm.

### Thermoluminescence measurements

Thermoluminescence (TL) was measured according to Cser and Vass (2007). Samples containing 30 µg chlorophyll *a* were exposed to a single actinic flash at -10 °C, then cooled to -40 °C. TL signals were recorded as the sample was heated at 20 °C min^− 1^ to 80 °C. When used, 60 µM DCMU was added before the actinic flash.

### Gel electrophoresis and western blotting

Thylakoid membrane isolation and blue-native polyacrylamide gel electrophoresis (BN-PAGE) were performed as described in Fagerlund et al. (2020). Samples containing 1.3 µg chlorophyll *a* were loaded onto precast 4–16% Bis-Tris gradient gels (Thermo Fisher Scientific, Waltham, MA, U.S.A.) and run at 4 °C. Proteins were transferred to a polyvinylidene difluoride membrane and probed with protein-specific antibodies for D1, D2, CP47 and CP43 (Agrisera, Vännäs, Sweden), followed by incubation with a secondary antibody (anti-rabbit IgG peroxidase (Sigma-Aldrich, St Louis, MO, U.S.A.)) and visualization by enhanced chemiluminescence (Eaton et al. 2014).

### ^35^S-methionine protein labeling

Samples at 10 µg mL^–1^ chlorophyll *a* in 40 mL were exposed to white light at ~ 2000 µmol photons m^–2^ s^–1^ for 45 min. Following the high-light exposure, 10 µCi mL^–1^of ^35^S-methionine (^35^S-Met) (PerkinElmer ) was added and 2 mL samples were collected at 10 min, 30 min and 120 min after the addition of label. The samples were centrifuged at 16 000×*g* for 1 min and the pellets resuspended in buffer containing 25 mM MES-NaOH (pH 6.5), 10 mM CaCl_2_, 10 mM MgCl_2_, 40% glycerol, and 1 tablet of protease inhibitor cocktail (Roche, Basel, Switzerland). Radiolabeled thylakoid membranes were isolated as described in Fagerlund et al. (2020) and membranes equivalent to 2 µg of chlorophyll *a* were solubilized in an equal volume of 0.5% dodecyl β-D-maltoside and placed on ice for 1 min. Samples were centrifuged at 16 000×*g* for 15 min at 4 ˚C and the supernatants mixed with 0.6% Serva Blue G250 (Serva, Heidelberg, Germany) before loading onto precast 4–16% Bis-Tris gradient gels (Thermo Fischer Scientific). The gels were run at 4 °C followed by 2D-gel electrophoresis. For 2D-gel electrophoresis, the desired individual lane following BN-PAGE was treated as described in Fagerlund et al. (2020) and proteins separated in the second dimension on a precast NuPAGE 12% Bis-Tris gel (Thermo Fisher Scientific). Autoradiograms were obtained by exposing film (Kodak, Rochester, NY, U.S.A.) in a Dupont Cronex intensifying screen lightning plus exposure cassette (Sigma-Aldrich) for 12–48 h at -80 °C.

## Results

### Photoautotrophic growth and accumulation of PS II monomers and dimers were similar in all strains

All five mutant strains: A240D, E241A, E242A, E242D and T243A grew photoautotrophically with similar growth rates to the control strain (Fig. S1). The BN-PAGE and the western blot indicated similar PS II dimers and monomers for all strains. There was increased unincorporated CP47 in the E241A, E242A, E242D and T243A mutants compared to the control. Unincorporated CP43 was increased in all the mutants except E242A, with the level in the T243A strain showing a marked increase compared to the other strains (Fig. S1).

### Replacing Ala240 with Asp or Glu241 with Ala had subtle impacts on PS II

Whole chain oxygen evolution rates in the presence of bicarbonate were similar in the A240D, E241A and control strains (Table 1). Oxygen evolution rates for the A240D mutant varied depending on the artificial electron acceptor used; with rates of ~ 70% and ~ 92% of the control in the presence of acceptor DMBQ or DCBQ, respectively (Table 1). In contrast, the E241A mutant exhibited similar rates to the control with either DMBQ or DCBQ (Table 1). Since both DMBQ and DCBQ are expected to bind to the Q_B_-binding site, these results suggest DMBQ binding was altered in the A240D strain (Kamada et al. 2023).

**Table 1:** Relative rates of oxygen evolution for the control, and the A240D, E241A, E242A, E242D and T243A mutants, in the presence of different electron acceptors

Electron acceptors	Oxygen evolution (µmol O_2_ (mg Chl)^−1^ h^− 1^)					
	Control	A240D	E241A	E242A	E242D	T243A
DMBQ	409 ± 39	283 ± 33	400 ± 40	311 ± 20	272 ± 21	339 ± 30
DCBQ	523 ± 12	480 ± 40	526 ± 27	442 ± 55	457 ± 46	312 ± 25
HCO_3_^−^	513 ± 23	495 ± 34	516 ± 43	490 ± 28	476 ± 43	545 ± 28

Errors displayed in the table are the standard errors calculated from the average of three independent experiments

**Fig. 2:**
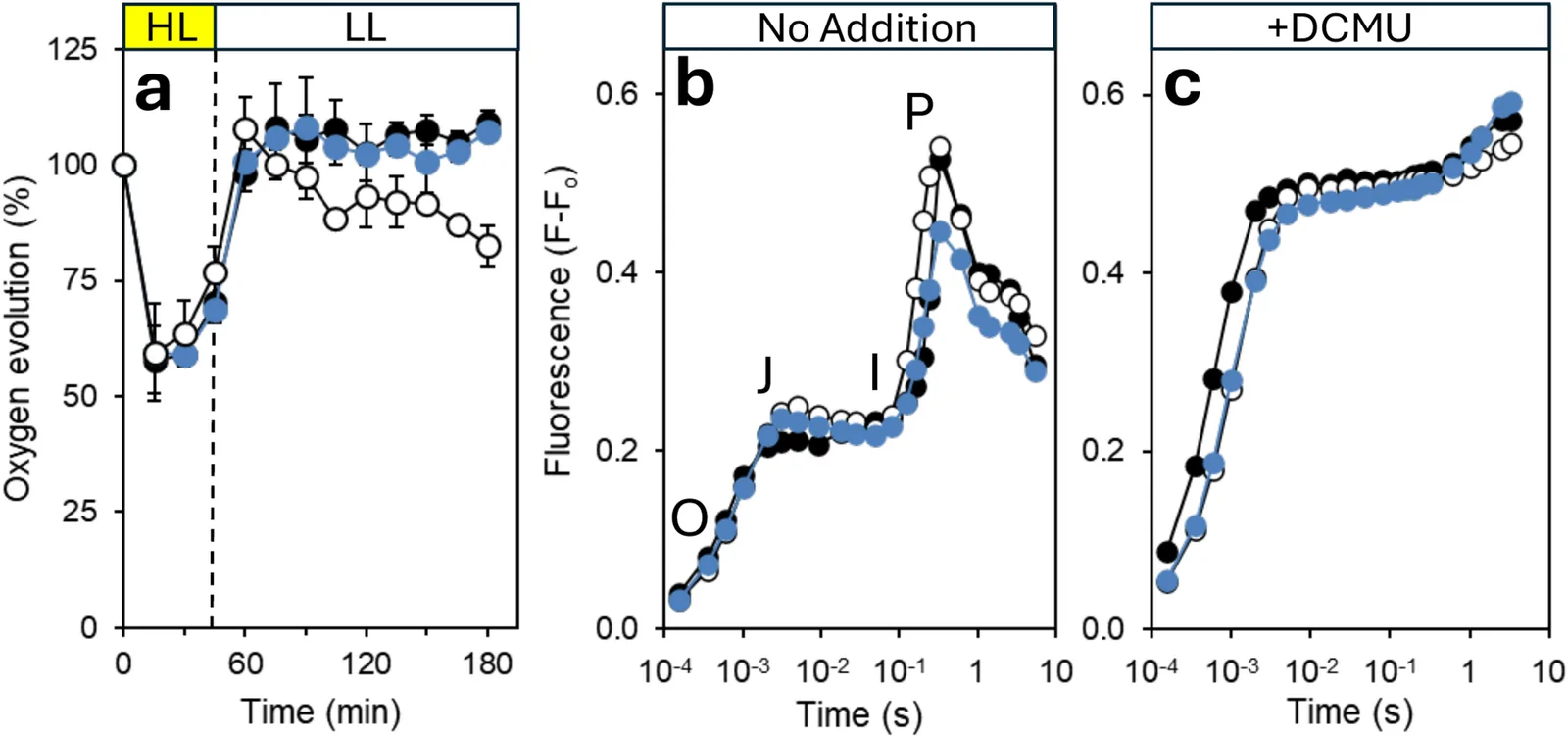
Steady-state electron transfer in the A240D and E241A strains is similar to control cells. (**a**) Oxygen evolution after exposure to high light (HL) at 2000 µmol photons m^–2^ s^–1^ for 45 min followed by low light (LL) 30 µmol photons m^–2^ s^–1^ for 135 min. Oxygen evolution was measured in the presence of DMBQ and K_3_Fe(CN)_6_. Error bars represent the standard error from three independent experiments. (**b**) Variable chlorophyll *a* fluorescence induction in the absence of DCMU. (**c**) Variable chlorophyll *a* fluorescence induction in the presence of DCMU. In panels b and c the data are the average of three independent experiments with only selected data points shown for clarity. Control (black filled circles), A240D (black empty circles), and E241A (blue filled circles)

The sensitivity of mutant cells to stress induced by high-intensity light was determined in the presence DMBQ. The proportional decrease in oxygen evolution rates during exposure to high light and subsequent recovery was similar for the A240D and E241A mutants and the control (Fig. 2a).

To further investigate active PS II centers, variable chlorophyll *a* fluorescence induction was measured. In the absence of DCMU, the control strain exhibited a typical O–J–I–P transient, starting from Fo (minimal fluorescence intensity) followed by the J peak (reflecting the amount of reduced Q_A_) and then the I inflection (reflecting PQ pool reduction) to a maximum P level which is equal to F_m_, the maximum fluorescence yield, when all Q_A_ is reduced (Stirbet and Govindjee 2011). In the absence of DCMU, the A240D and E241A strains showed a similar pattern to the control strain (Fig. 2b). The E241A mutant, however, exhibited a lower peak fluorescence yield. The variable fluorescence yield in the presence of DCMU is typically representative of the relative level of active PS II centers (Vass et al. 1999). Both the A240D and E241A strains showed a similar maximum or Fm level to the control, consistent with similar levels of active PS II centers (Fig. 2c). Additionally, low-temperature (77 K) fluorescence using 440 nm light to preferentially excite chlorophyll *a* resulted in similar emission spectra from the mutant and control strains (Fig. S2a). In addition, the three strains had similar emission spectra from low-temperature fluorescence using 580 nm excitation to preferentially excite the phycobilisome (Fig. S2b). These spectra are consistent with similar numbers of PS II centers in the three strains.

**Fig. 3:**
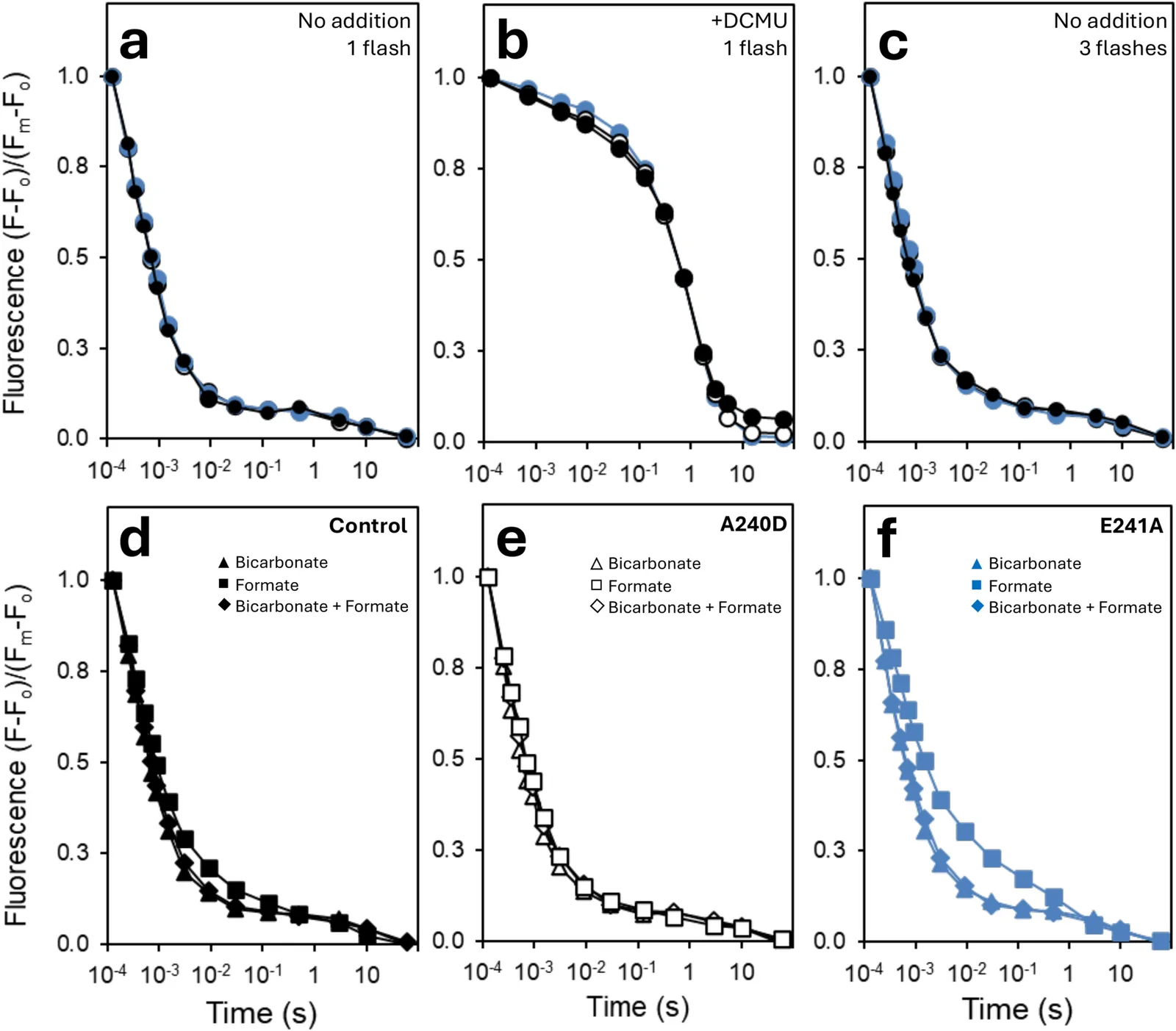
Decay of variable chlorophyll *a* fluorescence following one or three actinic flashes. (**a**) Variable fluorescence decay following a single-turnover actinic flash in the absence of DCMU. Control (black filled circles), A240D (black empty circles), and E241A (blue filled circles). (**b**) Variable fluorescence decay following a single-turnover actinic flash in the presence of DCMU. Symbols as in panel a. (**c**) Variable fluorescence decay following three saturating actinic flashes spaced at 200 ms intervals. Symbols as in panel a. (**d**) Variable fluorescence decay in control cells following a single turnover actinic flash in the presence of 25 mM formate (black filled squares), 15 mM bicarbonate (black filled triangles), or 15 mM bicarbonate and 25 mM formate (black filled diamonds). (**e**) Variable fluorescence decay in A240D cells following a single turnover actinic flash in the presence of 25 mM formate (black empty squares), 15 mM bicarbonate (black empty triangles), or 15 mM bicarbonate and 25 mM formate (black empty diamonds). (**f**) Variable fluorescence decay in E241A cells following a single turnover actinic flash in the presence of 25 mM formate (blue filled squares), 15 mM bicarbonate (blue filled triangles), or 15 mM bicarbonate and 25 mM formate (blue filled diamonds). Selected data points are shown for clarity. Data displayed are the average of at least three independent experiments

Electron transfer between the Q_A_ and Q_B_ electron acceptors of PS II can be followed by measuring the decay of chlorophyll *a* fluorescence after a single actinic flash. The chlorophyll *a* decay kinetics consist of a fast (µs) phase reflecting oxidation of Q_A_^**–**^ by bound Q_B_, (or Q_B_^**–**^ if present) an intermediate (ms) phase thought to arise from Q_A_^–^ oxidation in PS II centers where Q_B_ was not bound before the actinic flash and a slow (s) phase which corresponds to the back reaction with the S_2_ state of the OEC (Vass et al. 1999). In the A240D and E241A mutants, variable chlorophyll *a* fluorescence decay was slightly altered compared to the control following a single actinic flash in the absence DCMU with the fast component somewhat slower in the mutants (t_1/2_ control, 270 µs [amp 61%] vs. t_1/2_ A240D, 316 µs [amp 68%]; and t_1/2_ E241A, 322 µs [amp 64%] (Fig. 3a; Table 2). In the presence of DCMU variable chlorophyll *a* fluorescence decay was similar in the three strains (Fig. 3b; Table 2). To determine if the introduced substitutions impacted the exchange of PQH_2_ at the Q_B_-binding site the decay of variable fluorescence was also recorded after three single-turnover actinic flashes. The kinetic analysis for chlorophyll *a* fluorescence decay following three flashes showed the two mutants had a slower intermediate phase (t_1/2_ control, 3.0 ms [amp 27%] vs. t_1/2_ A240D, 7.0 ms [amp 24%]; and t_1/2_ E241A, 8.2 ms [amp 20%] (Fig. 3c; Table S2). An increase in the rate and amplitude for the slow component was also observed in all strains and likely arose, at least in part, due to the increased population of the S_0_ state of the OEC in the cells (Table S2).

**Table 2:** Kinetic analysis of the decay of chlorophyll *a* fluorescence following a single turnover actinic flash in the presence or absence of DCMU^a^

Strain	Treatment	Fast component		Intermediate component		Slow component	
		Rate (t_1/2_ = µs)	Amplitude (%)	Rate (t_1/2_ = ms)	Amplitude (%)	Rate (t_1/2_ = s)	Amplitude (%)
control	No treatment	270 ± 21.0	61.0 ± 3.0	2.4 ± 0.3	31.0 ± 3.0	5.8 ± 0.7	8.0 ± 1.0
	+DCMU			1.6 ± 0.2	10.0 ± 1.0	0.7 ± 0.1	90.0 ± 1.0
A240D	No treatment	316 ± 8.8	67.7 ± 0.3	2.6 ± 0.9	25.0 ± 0.4	5.3 ± 0.5	7.4 ± 0.7
	+DCMU			1.6 ± 0.0	11.8 ± 0.1	0.7 ± 0.0	88.2 ± 0.1
E241A	No treatment	322 ± 6.7	64.0 ± 0.6	2.5 ± 0.0	29.0 ± 0.7	5.2 ± 1.5	7.6 ± 1.2
	+DCMU			1.7 ± 0.2	8.4 ± 0.3	0.6 ± 0.0	91.6 ± 0.3
E242A	No treatment	346 ± 2.1	61.0 ± 1.5	7.0 ± 0.9	32.0 ± 1.3	2.0 ± 0.5	7.0 ± 0.2
	+DCMU			3.9 ± 1.5	8.5 ± 1.9	1.1 ± 0.1	92.0 ± 2.0
E242D	No treatment	377 ± 33.0	53.0 ± 0.4	3.0 ± 0.0	40.0 ± 0.5	7.0 ± 0.7	7.0 ± 0.0
	+DCMU			2.1 ± 0.2	8.9 ± 1.8	1.3 ± 0.0	91.0 ± 1.7
T243A	No treatment	412 ± 22.0	45.0 ± 3.0	3.0 ± 0.2	36.0 ± 4.0	6.0 ± 0.7	16.0 ± 1.7
	+DCMU			2.1 ± 0.1	11.0 ± 0.2	0.8 ± 0.0	89.0 ± 0.2

^a^Kinetic analyses were performed according to Vass et al. (1999). Data are the average of three independent experiments, and the standard error of the mean for the calculated rates and amplitudes is shown

Formate can displace bicarbonate from the NHI and lead to slow electron transfer from Q_A_^**–**^ to Q_B_ (Robinson et al. 1984; Eaton-Rye and Govindjee 1988a,b; Sedoud et al. 2011). As the fast phase of the fluorescence decay was slowed in the two mutants, reflecting slower oxidation of Q_A_^**–**^ by bound Q_B_, the decay of chlorophyll *a* fluorescence was measured following a single turnover actinic flash after the addition of either formate or bicarbonate, or both bicarbonate and formate (Fig. 3d-f). This resulted in a slight bicarbonate-reversible formate effect in control cells (Fig. 3d; Table S3), but A240D cells were relatively insensitive to either bicarbonate or formate addition (Fig. 3e; Table S3). In contrast, in the E241A mutant, formate slowed the decay of fluorescence in a bicarbonate-reversible manner (Fig. 3f; Table S3).

### Mutation of Glu242 or Thr243 decreased rates of oxygen evolution and the T243A mutant showed increased sensitivity to high light

Whole chain oxygen evolution rates in the presence of bicarbonate were similar in the E242A, E242D, T243A and control strains (mutants showed > 92% of the control rate; Table 1). Oxygen evolution rates with PS II-specific electron acceptors, however, were somewhat decreased and varied depending on the artificial electron acceptor used. In the presence of DMBQ, rates were ~ 76%, ~ 67%, and ~ 83% of the control for the E242A, E242D, T243A strains, respectively and in the presence of DCBQ, rates were ~ 85%, ~ 87%, and ~ 60% of the control for the E242A, E242D, T243A strains, respectively (Table 1). These observations suggest that binding of both DMBQ and DCBQ may have been altered in these strains since it has been noted that tighter binding of artificial electron acceptors at the Q_B_-binding site equates with lower rates of oxygen evolution (Kamada et al. 2023).

**Fig. 4:**
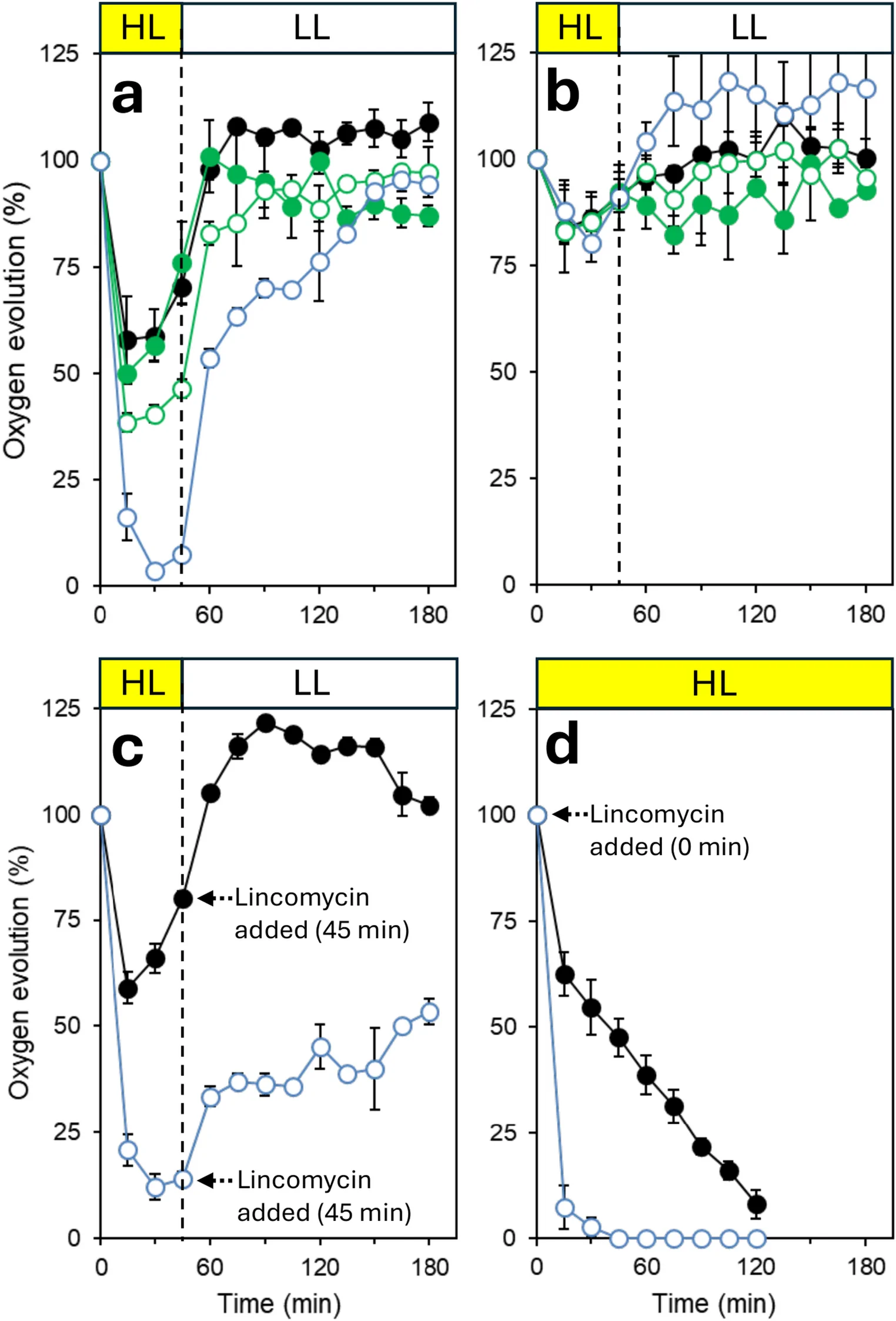
The effect of high-light exposure on oxygen evolution in E242A, E242D and T243A cells. (**a**) Oxygen evolution following incubation in high light (HL) (2000 µmol photons m^–2^ s^–1^) and recovery at low light (LL) (30 µmol photons m^–2^ s^–1^). Oxygen evolution was measured in the presence of DMBQ and K_3_Fe(CN)_6_. Control (black filled circles), E242A (green filled circles), E242D (empty green circles), and T243A (empty blue circles). (**b**) Oxygen evolution following incubation in high light and recovery under low light. Oxygen evolution was measured in the presence of bicarbonate. Symbols and light treatments as in panel a. (**c**) Oxygen evolution after incubation under high light and following the addition of lincomycin at the time of transfer to low light. Oxygen evolution was measured in the presence of DMBQ and K_3_Fe(CN)_6_. Control (black filled circles) and T243A (empty blue circles). (**d**) Oxygen evolution after incubation under high light in the presence of lincomycin added at the onset of the high-light treatment. Oxygen evolution was measured in the presence of DMBQ and K_3_Fe(CN)_6_, Symbols as in panel c. Error bars in all panels represent the standard error from three independent experiments

Exposure to high-intensity light (2000 µmol photons m^− 2^ s^− 1^) was performed to assess the vulnerability of the cells to photodamage. The E242A and control strains showed a similar proportional decrease in oxygen evolution rates during high-light treatment and subsequent recovery (Fig. 4a). The E242D strain showed increased sensitivity to high light with oxygen evolution reduced to ~ 40% of the initial rate (compared to approximately 60% in the control strain); however, in low light (30 µmol m^− 2^ s^− 1^) it recovered to ~ 90% of the initial rate. In contrast, the T243A mutant was unable to support oxygen evolution during the high-light exposure, and its recovery was slower than the control strain (Fig. 4a). Notably, whole-chain rates of oxygen evolution were sustained indicating that sufficient PS II centers remained even during the high-light exposure to enable the native quinone to support electron transfer, and that under the saturating light used for the measurement, the rate-limiting step of quinol oxidation was the same in all strains (Fig. 4b).

To examine the ability of the T243A mutant to recover from high-light treatment (Fig. 4a) and to support whole chain electron transfer rates like control cells (Fig. 4b), the protein synthesis inhibitor, lincomycin, was added. When lincomycin was added after the 45-min high-light treatment, the recovery of oxygen evolution during the low-light period was substantially impaired (Fig. 4c). In addition, when lincomycin was added at the start of an extended high-light treatment, the T243A cells were completely inactivated after only 15 min of high light, whereas it took a two-hour exposure to inactivate control cells (Fig. 4d).

To investigate the delayed recovery of the T243A mutant, the PS II repair mechanism was examined using ^35^S-Met incorporation following high-light exposure. Cells were exposed to high light for 45 min then ^35^S-Met was added. Thylakoids were extracted and the incorporation of labeled amino acids during the recovery stage was detected using BN-PAGE followed by two-dimensional SDS-PAGE. The autoradiograms of the two-dimensional protein analysis showed that by the end of the recovery period more label had been incorporated in the T243A mutant than in the control cells suggesting higher rates of repair occur in the mutant (Fig. S3) (Fagerlund et al. 2020; Biswas and Eaton-Rye 2022).

### Room temperature variable chlorophyll *a* fluorescence induction showed PS II centers were impaired in the E242A, E242D and T243A mutants

**Fig. 5:**
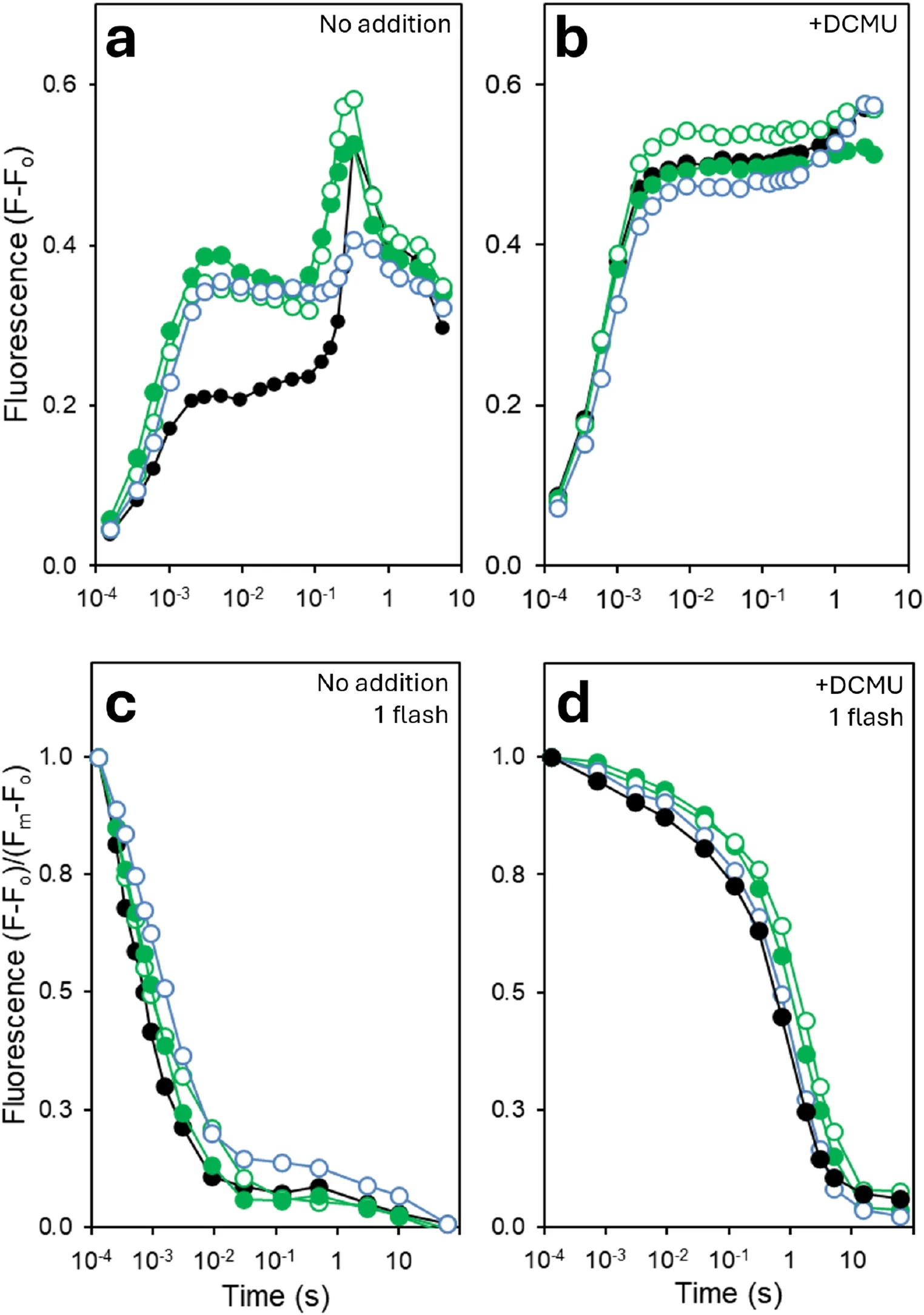
Variable chlorophyll *a* fluorescence from E242A, E242D and T243A cells. (**a**) Variable chlorophyll fluorescence induction in the absence of DCMU. Control (black filled circles), E242A (green filled circles), E242D (green empty circles) and T243A (blue empty circles). (**b**) Variable chlorophyll fluorescence induction in the presence of DCMU. Symbols as in panel a. (**c**) Decay of chlorophyll fluorescence after a single actinic flash in the absence of DCMU. Symbols as in panel a. (**d**) Decay of chlorophyll fluorescence after a single actinic flash. in the presence of DCMU. Symbols as in panel a. All data are the average of three independent experiments

To further investigate the effects resulting from mutations at positions D2:Glu242 and D2:Thr243 we measured variable chlorophyll *a* fluorescence induction. In the absence of DCMU all strains exhibited an O–J–I–P induction curve; however, compared to the control, the E242A, E242D and T243A mutants had an increased J level indicative of slowed electron transfer from Q_A_^**–**^ (Fig. 5a). The T243A cells exhibited a reduced P level relative to control and E242A cells; however, in the presence of DCMU, that blocks forward electron transfer from Q_A_^**–**^, the F_m_ reached by the control and the E242A and T243A mutants was similar (Fig. 5b). In both the absence and presence of DCMU the maximum fluorescence level reached by E242D cells was elevated, consistent with these cells having a greater fraction of PS II in state 1 (i.e., preferential energy transfer to PS II) (Calzadilla and Kirilovsky 2020).

The variable fluorescence yield of the E242A, E242D and T243A strains, following addition of DCMU, was similar to the yield observed in the control, consistent with comparable numbers of active PS II centers in all strains (Fig. 5b). These results were consistent with the low-temperature fluorescence emission spectra that were collected following excitation at 440 nm and 580 nm where the emission spectra for the mutants were also like the control (Fig. S4).

### Variable chlorophyll *a* fluorescence decay was altered in the E242A, E242D and T243A mutants

Measurements of the decay of variable fluorescence revealed all three mutant strains had a slower fast component compared to the control, reflecting altered oxidation of Q_A_^−^ by bound Q_B_, this was particularly marked in the T243A strain (t_1/2_ control, 270 µs [amp 61%] vs. t_1/2_ E242A, 346 µs [amp 61%]; t_1/2_ E242D, 377 µs [amp 53%]; and t_1/2_ T243A 412 µs [amp 45%] (Fig. 5c; Table 2). The half time for the intermediate phase of the E242A strain was slower compared to the control (t_1/2_ control, 2.4 ms [amp 31%] vs. t_1/2_ E242A, 7.0 ms [amp 32%]) whereas the E242D and T243A strains had a similar intermediate phase to the control (Fig. 5c; Table 2). The slow phase was faster in the E242A strain compared to the control (t_1/2_ control, 5.8 s [amp 8%] vs. t_1/2_ E242A, 2.0 s [amp 7%] (Fig. 5c; Table 2)). The rates of the slow component for E242D and T243A cells were, however, similar to the control strain, although the amplitude increased to 16% in the T243A strain.

In the presence of DCMU, the largest difference was observed in the fast component of the E242A mutant when compared to the control, but this component was modified to a lesser extent in the E242D and T243A strains (t_1/2_ control, 1.6 ms [amp 10%] vs. t_1/2_ E242A, 3.9 ms [amp 9%]; t_1/2_ E242D, 2.1 ms [amp 9%]; and t_1/2_ T243A, 2.1 ms [11%] (Fig. 5d; Table 2)). The slow component was slightly modified in the E242A mutant and the E242D strain, while this component in the T243A strain was similar to the control (Fig. 5d; Table 2).

**Fig. 6:**
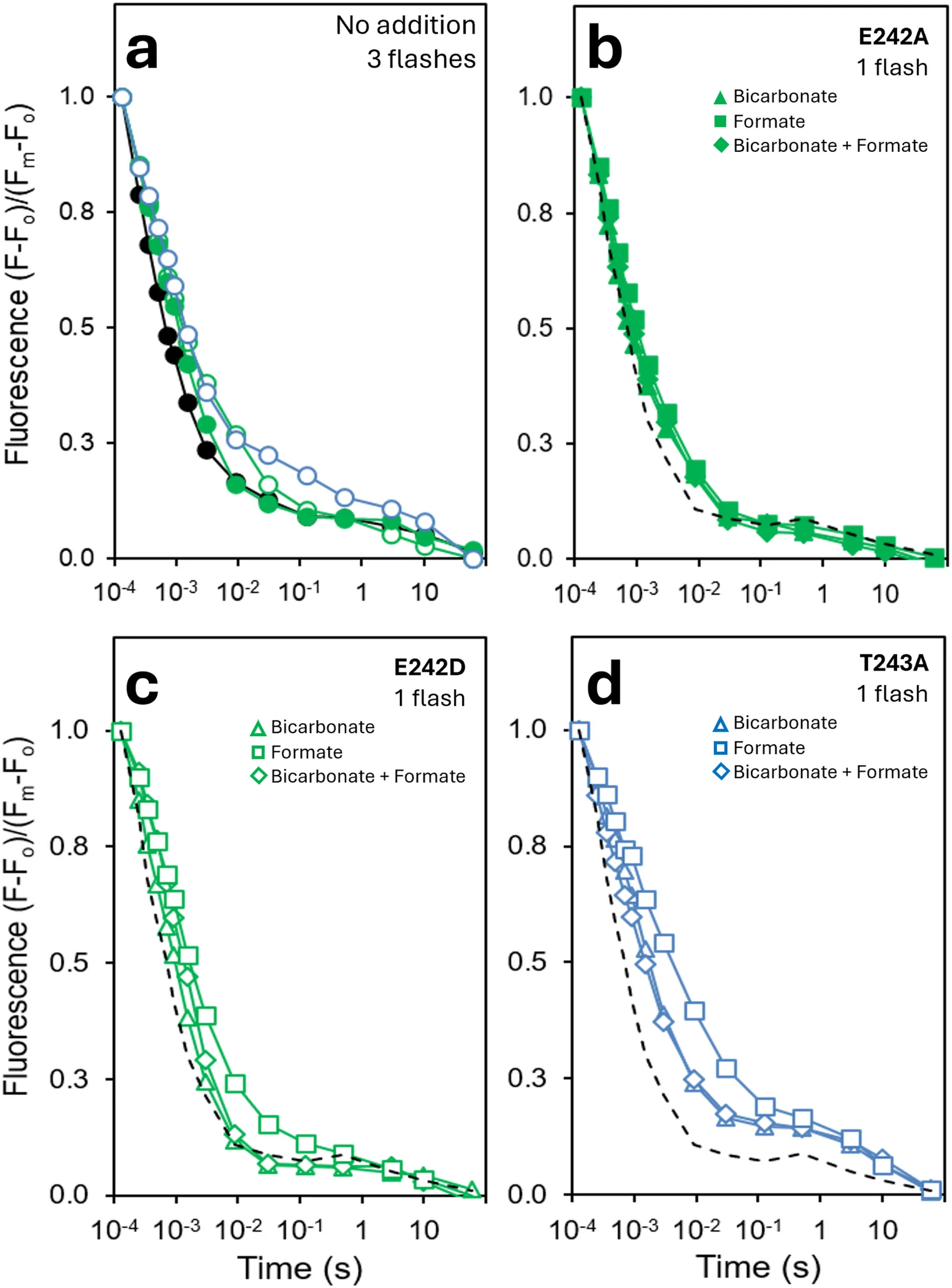
The decay of chlorophyl fluorescence following three actinic flashes or after a single actinic flash in the presence of formate and bicarbonate in E242A, E242D and T243A cells. (**a**) Decay of variable fluorescence after three actinic flashes spaced at 200 ms. Control (black filled circles), E242A (green filled circles), E242D (green empty circles) and T243A (blue empty circles). (**b**) Variable fluorescence decay in E242A cells following a single turnover actinic flash in the presence of 25 mM formate (green filled squares), 15 mM bicarbonate (green filled triangles), or 15 mM bicarbonate and 25 mM formate (green filled diamonds). (**c**) Variable fluorescence decay in E242D cells following a single turnover actinic flash in the presence of 25 mM formate (green empty squares), 15 mM bicarbonate (green empty triangles), or 15 mM bicarbonate and 25 mM formate (green empty diamonds). (**d**) Variable fluorescence decay in T243A cells following a single turnover actinic flash in the presence of 25 mM formate (blue empty squares), 15 mM bicarbonate (blue empty triangles), or 15 mM bicarbonate and 25 mM formate (blue empty diamonds). Selected data points are shown for clarity. The dashed black line in panels b–d indicates the fluorescence decay from Fig. 5c for the untreated control to aid comparison. Data displayed are the average of at least three independent experiments

Chlorophyll *a* fluorescence relaxation following three saturating actinic flashes was also performed. The half-time of the intermediate ms phase following three flashes was slower than control in the E242A strain and the amplitude of the fast phase was decreased relative to the control (Fig. 6a, Table S2). The millisecond phase was also slower in the E241A mutant (Table S2). The E242D and T243A mutants, had similar traces for the fluorescence decays following 1 flash or 3 actinic flashes with T243A exhibiting the most impaired decay (Fig. 6a, Table S2).

The effect of formate on the fluorescence decay after one actinic flash was also investigated in the E242A, E242D and T243A strains (Fig. 6b-d). There was little effect of formate addition in the E242A strain, and no change was observed when bicarbonate was added. In contrast, formate addition slowed electron transfer in E242D and T243A cells with bicarbonate addition able to recover the decay to near control rates in the E242D mutant and to partially restore the decay in the T243A mutant (Table S3).

### Thermoluminescence was altered in the E242A, E242D and T243A mutants

**Fig. 7:**
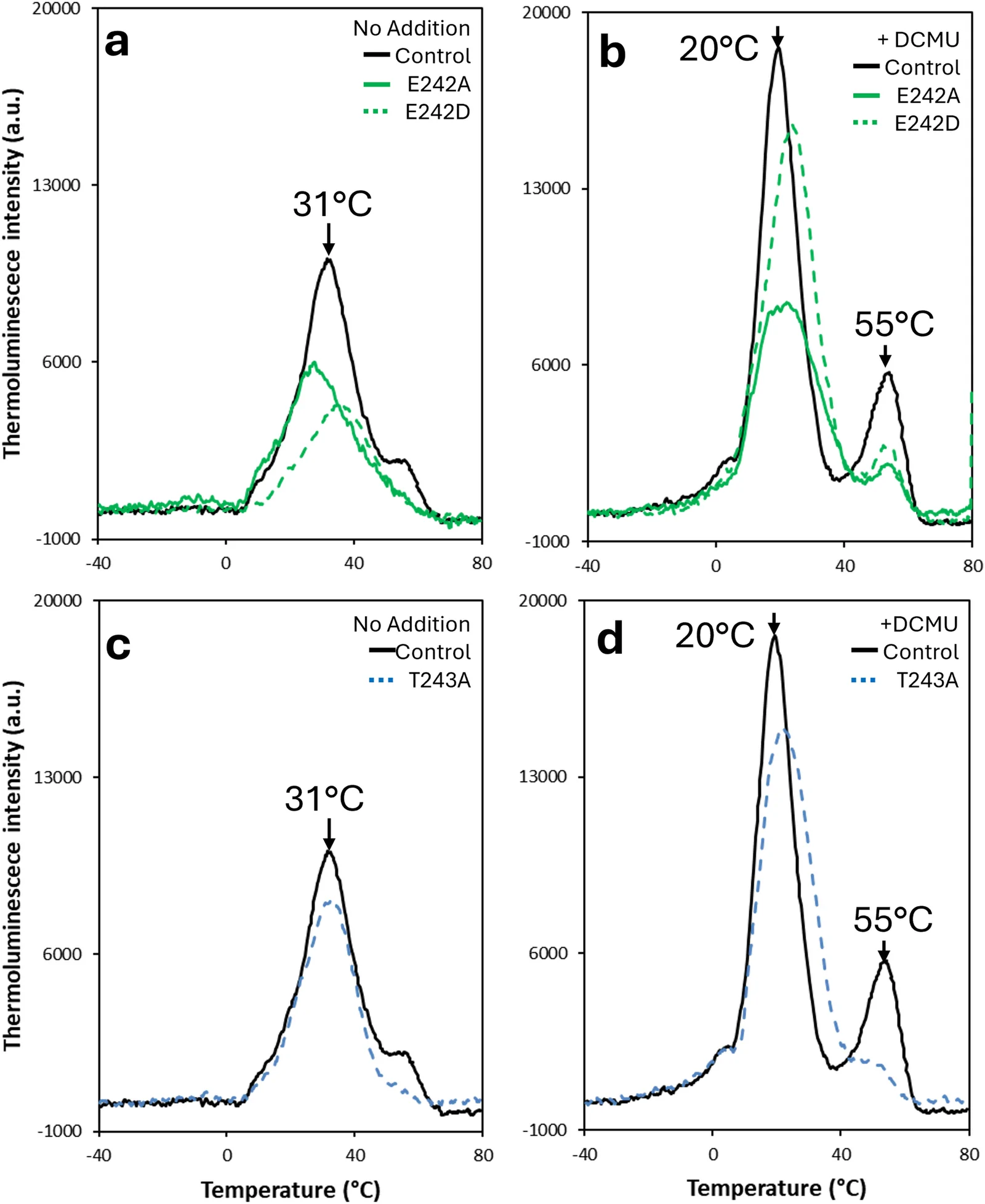
Thermoluminescence (TL) analysis of the E242A, E242D and T243A mutants. (**a**) TL curves in the absence of DCMU. Control (black line), E242A (green line) and E242D (green dashed line). (**b**) TL curves in the presence of DCMU. The strains are the same as panel a. (**c**) TL curves in the absence of DCMU. Control (black line), T243A (blue dashed line). (**d**) TL curves in the presence of DCMU. The strains are the same as panel c. For all measurements, cells were excited by a single actinic flash at -10 ℃ and TL emission was recorded in the temperature range of -40 ℃ to 80 ℃. Data displayed are from the average of three independent experiments

To further investigate the impact of the introduced substitutions at D2:Glu242 and D2:Thr243 thermoluminescence (TL) measurements were performed (Fig. 7a-d). After a single turnover actinic flash three distinct TL bands originate from recombination reactions (Ducruet and Vass 2009). The B band from S_2_Q_B_^**•–**^ (or S_3_Q_B_^**•–**^) recombination, the Q band resulting from S_2_Q_A_^**•–**^recombination and the C band representing recombination between Q_A_^•**–**^ (or Q_B_^•**–**^) with Y_D_(ox) (i.e., the neutral radical of Tyr160 of the D2 protein). The addition of DCMU is required to observe the Q band, whereas the C band is observed in the presence or absence of DCMU (Cser and Vass 2007).

The temperature of the B band in the E242A mutant decreased compared to the control by approximately 4 °C whereas the B band in the E242D strain remained similar (Table 3). The intensity of the emission was also reduced in the mutants (Fig. 7a). In the presence of DCMU the Q band for the E242A strain was increased above the control (from ca. 20 °C to 23 °C) and again the intensity was reduced, whereas the E242D cells also showed an increase to ~ 23 °C but with a smaller decrease in the signal (Fig. 7b; Table 3). The C-band for this pair of mutants was only observed in the presence of DCMU and exhibited a decrease in amplitude relative to control cells.

In the T243A mutant, the B band remained at a similar temperature to the control with no clear C band, but in the presence of DCMU the Q band shifted slightly from 20 °C to 21 °C and a small C band was present (Fig. 7c, d; Table 3).

## Discussion

### Mutations targeting Ala240 to Thr243 in the conserved Ala240–Asn250 region of the D2 DE-loop did not disrupt PS II assembly or prevent photoautotrophic growth

The bicarbonate cofactor that serves as a bidentate ligand to the NHI of PS II is stabilized by the DE-loops of both the D1 and D2 proteins (Umena et al. 2011). In particular, the D1 sequence ^242^E**EETY**NIVAAH^252^ and the D2 sequence ^240^A**EETY**SMVTAN^250^ directly contribute by providing Tyr ligands (D1-Tyr246 and D2-Tyr44) to bicarbonate and in participating in a hydrogen bond network that is directly involved in the protonation of the reduced Q_B_^**–**^ semiquinone and the doubly reduced Q_B_^2**–**^ (Saito et al. 2013; Nihara et al. 2025). In this study, we targeted the conserved ^240^AEET^243^ sequence of the D2 protein by constructing the A240D, E241A, E242A, E242D and T243A mutants in *Synechocystis* 6803. All these mutants grew photoautotrophically and assembled PS II (Fig. S1); however, a noticeable accumulation of unassembled CP43 was evident in the T243A strain. In contrast, mutations targeting D2-Met246 and D2-Asn250, that are also located in the ^240^Ala–Asn^250^ conserved region of D2, impaired PS II activity. The D2-M246A mutant had slowed photoautotrophic growth and altered PS II assembly, while the D2-N250A strain together with the D2-M246A mutant showed slowed electron transfer from Q_A_^**–**^ to Q_B_ with the D2-N250A strain exhibiting a down shift in the redox potential of the Q_A_/Q_A_^**–**^ couple. Hence, D2-Met246 and D2-Asn250 displayed key roles in the operation of the quinone-iron acceptor complex influencing both Q_A_ and bicarbonate binding (Zhong et al. 2025). Similarly, the D2-Y244A mutant of *Synechocystis* 6803 exhibited slowed electron transfer from Q_A_^**–**^ to Q_B_ and impaired PS II assembly while the D1-Y246A strain showed pronounced disruption of Q_A_^**–**^ to Q_B_ electron transfer, but this mutant did assemble PS II levels comparable to control cells (Forsman et al. 2019a; Khaing et al. 2022).

### Mutations targeting Glu242 in the D2 ^241^EETY^244^ motif were less deleterious than mutations of Glu244 within the D1 ^243^EETY^246^ motif

The DE loop of the D2 protein (residues 222–262) contributes to the Q_A_-binding environment and the DE loop of the D1 protein (residues 233–266) contributes to the Q_B_-binding environment (Gisriel et al. 2022b). Notably, the D2 ^241^EETY^244^ sequence is conserved in D1 (^243^EETY^246^) and found throughout oxygenic photoautotrophs (Zhong et al. 2025). Mutation of D1-Glu244 to Ala in *Synechocystis* 6803 resulted in the D1-E244A strain exhibiting weakened Q_B_ binding leading to impaired photoautotrophic growth which, along with the phenotype of the D1-Y246A strain, suggests mutations in the EETY motif in the vicinity of the Q_B_-binding site are more disruptive to electron transfer from Q_A_^**–**^ to Q_B_ than corresponding mutations in the D2 ^241^EETY^244^ sequence (Forsman et al. 2019a). Nevertheless, the decay of chlorophyll fluorescence after a single turnover flash in D2-E242A cells indicated an approximate three-fold increase in the millisecond component and the slow seconds component was consistent with a shift in the equilibrium for the sharing of an electron between Q_A_ and Q_B_ towards Q_A_^**–**^ (Fig. 5c; Table 2). Additionally, the extent of impairment of the fluorescence decay in the D2-E242A mutant remained similar after three turnover flashes spaced at 200 ms (Fig. 6a; Table S2); and, under steady-state conditions, an elevated J level was observed in fluorescence induction measurements, and rates of oxygen evolution were diminished (Fig. 5a; Table 1).

Generally, under steady-state conditions, the D2-E242D cells were slightly more impaired than the D2-E242A mutant (e.g., Fig. 4a; Table 1), despite the charge provided by Asp, although the millisecond and seconds components of the fluorescence decay after a single flash more closely resembled the situation in control cells (Table 2). Likewise, the TL B-band was shifted to a lower value in E242A cells but remained similar to the control strain in E242D cells, indicative of a more negative Q_B_/Q_B_^**–**^ redox potential in the E242A mutant (Fig. 7a; Table 3). In contrast, the introduction of Ala at the D1-Glu244 position resulted in a positive shift in the Q_A_/Q_A_^**–**^ redox potential and this was accompanied by an increased tolerance to high light (Forsman et al. 2019a). However, the sensitivity towards high light observed in the D2-E242A and D2-E242D mutants remained similar to that of the control cells (Fig. 4a) even though our data are also consistent with a slight increase in the Q_A_/Q_A_^**–**^ redox potential in these strains (Figs. 5d and 7; Tables 2 and 3).

**Table 3:** Thermoluminescence temperature maxima for the Q-band and B-band in *Synechocystis* sp. PCC 6803 mutants with substitutions at Glu242 and Thr243 of the D2 protein

Strain	Q band (°C)	B band (°C)
Control	19.6	31.3
E242A	22.6	27.7
E242D	23.3	32.6
T243A	21.2	32.3

The experiment was repeated three times

Two putative channels connecting the bicarbonate-binding site to the NHI with the cytosol were identified using the program CAVER (Fig. 1) and correspond to water channels A and B identified using Molecular Dynamics (MD) simulations (Sirohiwal and Pantazis 2022). Channel A also corresponds to the channel proposed to act in CO_2_ exchange between the NHI and the cytosol by Sugo and Ishikita (2022). D1-Glu244 is located within Channel A and has a putative hydrogen bond with the water A506 (see Fig. 1) that is conserved in *Thermosynechococcus vestitus* (Hussein et al. 2024) and *Thermostichus vulcanus* (Suga et al. 2015). Notably water A506 is hydrogen-bonded to water D572 which in turn forms a hydrogen bond with D2-Glu242, and, in addition, water D572 has a conserved interaction with the backbone carbonyl of D2-Glu241 via water D581 (Fig. 1). As noted above, despite this interconnected hydrogen bond network, electron transfer from Q_A_^**–**^ to Q_B_ is more impaired in the D1-E244A mutant than in the D2-E242A mutant (Forsman et al. 2019a and Fig. 5c).

The MD simulations using PS II from *T. vulcanus* (PDB: 3WU2) have indicated that both D1-Glu244 and D2-Glu242 interact with D2-Lys264 through a triple salt bridge that would be disrupted in the D1-E244A and D2-E242A mutants (Fantuzzi et al. 2022; Sirohiwal and Pantazis 2022). In addition, water D564 in *Synechocystis* 6803 (Fig. 1), that is located outside of channel B but conserved in *T. vestitus* (Hussein et al. 2024), bridges D2-Glu242 and D2-Lys264 potentially adding flexibility to the interaction. The presence of the D2-Glu242 and D2-Lys264 salt bridge would appear to restrict water exchange between Channel B and the NHI while the presence of water D564 appears to be associated with the open configuration whereby water access is favored (Fig. S5). Hence, it appears that D2-Lys264 can be present in either an open or closed configuration. Since D2-Lys264 forms a hydrogen bond with bicarbonate, the modified interaction between D2-Glu242 and D2-Lys264, resulting from the presence or absence of the bridging water, may influence the stability of the bicarbonate ligation to the NHI, as mutations targeting D2-Lys264 disrupt both PS II assembly and electron transfer (Khaing and Eaton-Rye 2023).

It has also been proposed that the bicarbonate affinity for the NHI may be decreased in the D1-E244A mutant relative to control cells because this mutant exhibited an enhanced tolerance to high light (Forsman et al. 2019a, b). Conditions resulting in the accumulation of Q_A_^**–**^ (such as high-light-induced stress) lead to the dissociation of bicarbonate and the concomitant increase in the redox midpoint potential of the Q_A_/Q_A_^**–**^ couple, such that direct recombination with P680^+^ is favored reducing the possibility of ^1^O_2_ formation via ^3^Chl formation (Brinkert et al. 2016; Sugo and Ishikita 2022). It is possible that the more pronounced inhibition of electron transfer from Q_A_^**–**^ to Q_B_ seen in D1-E244A cells, over that observed in the D2-E242A mutant, arises from both reduced bicarbonate binding and reduced Q_B_ binding.

Formate addition is known to displace bicarbonate, leading to slow electron transfer from Q_A_^**–**^ to Q_B_ and impaired quinone/quinol exchange at the Q_B_-binding site (Robinson et al. 1984; Sedoud et al. 2011; Forsman et al. 2019a). In the D1-E244A mutant, the impaired electron transfer from Q_A_^**–**^ to Q_B_ was insensitive to either the addition of bicarbonate or formate, possibly because the access of bicarbonate or formate to the NHI via Channel A in the D1-E244A mutant was prevented. Similarly, although the extent of the impaired Q_A_^**–**^ to Q_B_ electron transfer was less than seen in the D1-E244A cells, the D2-E242A cells were also insensitive to bicarbonate and formate addition (Fig. 6b); however, the D2-E242D cells were impaired in a bicarbonate-reversible manner when formate was added (Fig. 6c), suggesting that the preserved negative charge in the mutant (and by implication the preserved interaction with D2-Lys264) is required to allow access to the NHI. That the impaired Q_A_^**–**^ to Q_B_ electron transfer in both the D1-E244A and D2-E242A mutants does not respond to the addition of bicarbonate or formate may be explained by both Channel A and Channel B coalescing in the vicinity of bicarbonate (Fig. 1 and Fig. S5). In contrast to the D1-E244A mutant, however, the D2-E242D strain exhibited increased susceptibility to high-light-induced photodamage (Fig. 4a).

### The relative importance of Ala240 and Glu241 in the D2 ^240^AEET^243^ sequence

D2-Glu241 has a counterpart in D1-Glu243 in the conserved EETY sequence of the proteins. Interestingly, both D1-E243K and D1-E242Q mutants exhibited phenotypes similar to control or wild-type cells (Ohad and Hirschberg 1992; Mäenpää et al. 1993, 1995). The sensitivity of these mutants to formate addition was not reported; however, these previous studies did establish that the triple D1-∆EEE^242−4^ mutant and a related triple Glu deletion mutant, that also included a Gln to His substitution at the D1-Gln241 position, had impaired photoautotrophic growth and reduced rates of oxygen evolution. The latter triple Glu deletion mutant was also insensitive to formate addition (Kless et al. 1994; Mäenpää et al. 1995).

The corresponding position to D1-Glu242 is Ala240 in D2 and the D2-A240D mutant closely resembled control cells but differed to the control in being relatively insensitive to formate and bicarbonate additions (Figs. 2 and 3 and Tables S1 and S2). In contrast, the D2-E241A mutant exhibited Q_A_^**–**^ to Q_B_ electron transfer that was sensitive to formate in a manner resembling the sensitivity of the control (Fig. 3 and Tables S1 and S2). The D2-A240D and D2-E241A cells did, however, exhibit a slowed variable fluorescence rise in the presence of DCMU that was not observed in the D2-E242A mutant (cf. Figures 2c and 5b) which might reflect altered DCMU binding in the D2-A240D and D2-E241A cells.

### A putative role of D2-Glu241 in PS II biogenesis

In the RC47 pre-assembly complex of *T. vestitus* and *T. vulcanus*, D2-Glu241 is bound to the NHI instead of bicarbonate (Xiao et al. 2021; Zabret et al. 2021). In these organisms, the configuration whereby D2-Glu241 acts as a monodentate ligand to the NHI occurs in the presence of the Psb28 assembly factor which displaces the DE loops of both D1 and D2, additionally resulting in the Q_B_-binding site being unoccupied. A similar interaction of D2-Glu241 with the NHI was observed in an apo-PSII complex, lacking the lumenal extrinsic subunits and an intact OEC, from far-red-light grown *Synechococcus* sp. PCC 7335; however, in this organism D2-Glu241 bound to the NHI in the absence of Psb28 (Gisriel et al. 2022a). While conservation of the D2-Glu241:NHI interaction suggests a physiological role, our D2-E241A mutant was found to be like control cells (Figs. 2 and 3a-c), albeit with a slightly increased susceptibility to formate addition (Fig. 3d-f. and Table S3). Similarly, a D2-E241Q mutant constructed in *Chlamydomonas reinhartdii* exhibited a phenotype that was similar to that of the control strain (Fu et al. 2017). It remains possible, however, that under different environmental conditions the D2-Glu241:NHI interaction may be more important, for example, by preventing oxygen from binding to the NHI and thereby preventing the formation of light-driven O_2_^**–**^ production as observed in Fantuzzi et al. (2022). It may also be the case that the D2-Glu241:NHI interaction assists in preventing premature bicarbonate binding.

### Symmetrical D2-Thr243:D1-Arg269 and D1-Thr245:D2-Arg265 interactions in PS II influence the susceptibility to photodamage

The hydroxyl of D2-Thr243 forms a salt bridge (or hydrogen bond) with the Nε nitrogen of D1-Arg269 while the hydroxyl of D1-Thr245 (in the conserved ^243^EETY^246^ sequence of D1) forms a salt bridge (or hydrogen bond) with the Nη nitrogen of D2-Arg265 (Fig. S6) (Zhong et al. 2026). In turn, D2-Arg265 interacts with the D2-His268 residue that is a ligand to the NHI and D1-Arg269 interacts with the D1-His272 NHI ligand (Fig. S6). Mutations targeting D2-Arg265 indicated that the D2-Arg265/D2-His268/NHI interaction(s) contribute to stabilizing bicarbonate binding and facilitate forward electron transfer between Q_A_ and Q_B_ and the exchange of PQH_2_ between the Q_B_-binding site and the PQ pool in the thylakoid membrane. The investigated mutants (D2-R265A and D2-R265D) were also highly sensitive to photodamage (Zhong et al. 2026).

The D2-T243A mutant was the most impaired among the mutants constructed targeting the conserved D2 ^240^AEET^243^ sequence. The D2-T243A strain exhibited an increased fraction of centers undergoing a back reaction with the S_2_ state of the OEC in the absence of DCMU (Fig. 5c; Table 2). The D2-T243A strain was also the most sensitive to formate addition (Fig. 6 and Table S3) and the D2-T243A strain was the most sensitive to high-light-induced photodamage (Fig. 4). Since it has been shown in *C. reinhardtii* that the introduction of a glycine in place of D1-Arg269 disrupted both assembly of the OEC and forward electron transfer between Q_A_ and Q_B_ (Hutchison et al. 1996; Xiong et al. 1997), the deleterious phenotype of the D2-T243A mutant likely arises through disruption of the D2-Thr243/D1-Arg269 interaction and consequential perturbation of the D1-His272 coordination of the NHI. The relative importance of D1-Thr245 and the corresponding D2-Thr243, however, is uncertain as no mutations targeting D1-Thr245 have been reported.

## Supplementary Information

Below is the link to the electronic supplementary material.

Supplementary Material 1

## Data Availability

Data is provided within the manuscript or supplementary information.

## Declarations

### Accession Code

Photosystem II complex from *Synechocystis* sp. PCC 6803 (PDB 7N8O).
