# Supplementary material for: The contribution of the 240Ala:Glu:Glu:Thr243 sequence in the DE-loop of D2 to the acceptor side of Photosystem II
Source: Photosynth Res. 2026 Jun 16;164(4):34. doi: 10.1007/s11120-026-01222-4 (PMC13272216; doi:10.1007/s11120-026-01222-4)
Supplement: Supplementary file 1 — Supplementary Material 1 [file 11120_2026_1222_MOESM1_ESM.docx]

## Supplementary Information

**The contribution of the ^240^Ala:Glu:Glu:Thr^243^ sequence in the DE-loop of D2 to the acceptor side of Photosystem II**

**Ei Phyo Khaing^1^ • Tina C. Summerfield^2^ • Jack A. Forsman^1^ • Imre Vass^3^ • Priyanka Pradeep Patil^3,4^ • Julian J. Eaton-Rye^1*^**

^1^Department of Biochemistry, University of Otago, P.O. Box 56, Dunedin 9054, New Zealand

^2^Department of Botany, University of Otago, P.O. Box 56, Dunedin 9054, New Zealand

^3^Institute of Plant Biology, HUN-REN, Biological Research Center, Szeged, Hungary

^4^Doctoral School of Biology, Faculty of Science and Informatics, University of Szeged, Szeged, Hungary

***Corresponding Author**

Julian J. Eaton-Rye — Department of Biochemistry, University of Otago, Dunedin 9054, New Zealand; orcid.org/0000-0003-4137-8838

Email: [julian.eaton-rye@otago.ac.nz](mailto:julian.eaton-rye@otago.ac.nz)

**CONTENTS**

| Page S3  Page S4  Page S5  Page S6  Page S7  Page S8  Page S9 | **Fig. S1** Photoautotrophic growth and blue-native (BN) PAGE followed by western blot analysis  **Fig. S2**  Low-temperature (77 K) fluorescence emission spectra for the control and the A240D and E241A mutants  **Fig. S3** Analysis of photodamage and recovery in isolated thylakoid membranes  **Fig. S4** Low-temperature (77 K) fluorescence emission spectra for the control and the E242A, E242D and T243A mutants  **Fig. S5** Overlay of the DE-Loop regions of the D2 protein in the vicinity of D2-Lys264 from *Thermostichus vulcanus* (PDB: 3WU2) and *Synechocystis* sp. PCC 6803 (PDB: 7N8O)  **Fig. S6** Location of D2-Arg265 and D1-Arg269 in proximity to the non-heme iron and bicarbonate-binding environment of the acceptor side of PS II  **Table S1** Primer sequences used in QuikChange site-directed mutagenesis of the D2 protein to make the A240D, E241A, E242A, E242D and T243A mutants |
| --- | --- |
| Page S10 | **Table S2** Kinetic analysis of the decay of chlorophyll *a* fluorescence following three saturating actinic flashes spaced at 200 ms intervals in the A240D, E241A, E242A, E242D and T243A mutants |
| Page S11 | **Table S3** Kinetic analysis of the decay of chlorophyll *a* fluorescence after a single turnover flash for cells treated with 25 mM formate, 15 mM bicarbonate, or 15 mM bicarbonate and 25 mM formate in the in the A240D, E241A, E242A, E242D and T243A mutants |

Page S12 **References**


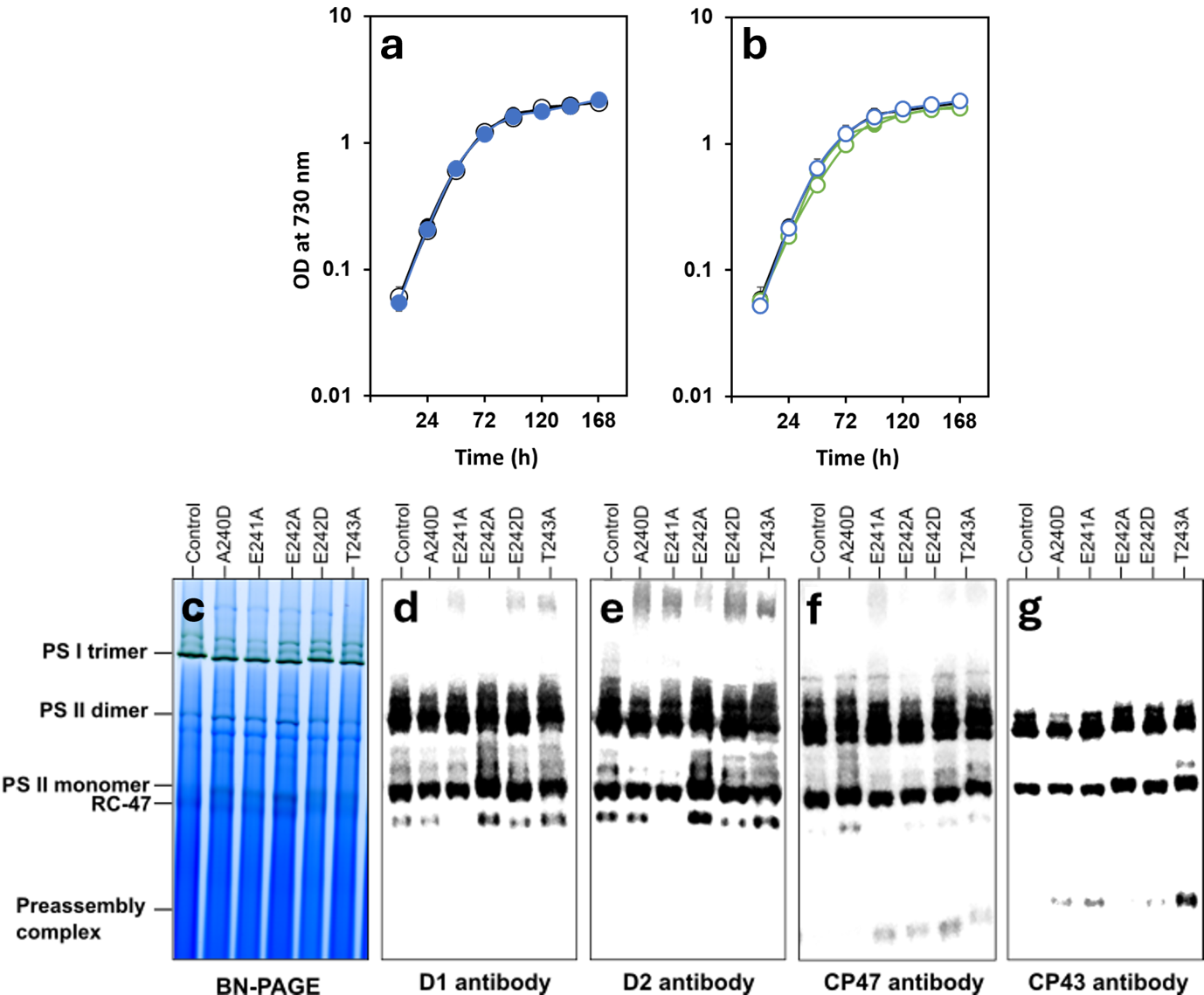


**Fig. S1** Photoautotrophic growth and blue-native (BN) PAGE followed by western blot analysis. **a** Photoautotrophic growth curve of control (black filled circles), A240D (black empty circles) and E241A (blue full circles). **b** Photoautotrophic growth curve of E242A (green full circles), E242D (green empty circles) and T243A (blue empty circles). The error bars represent the standard error from three independent experiments. **c** BN-PAGE gel. **d** Western blot with an antibody against D1. **e** Western blot with an antibody against D2. **f** Western blot with an antibody against CP47. **g** Western blot with an antibody against CP43. The BN-PAGE gel and western blots are labeled with the name of the strain above the corresponding lane. The experiment was repeated three times with similar results.


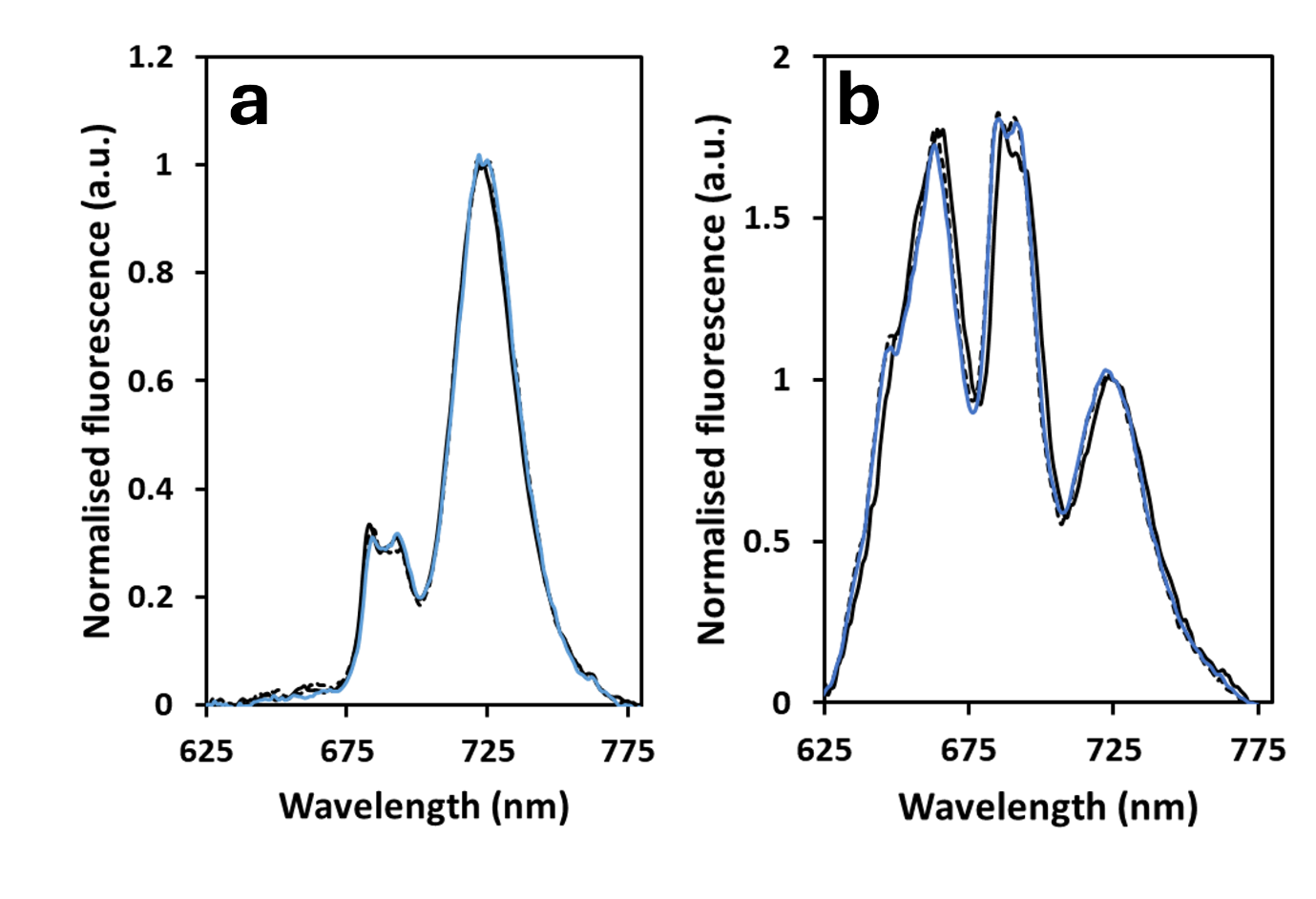


**Fig. S2**  Low-temperature (77 K) fluorescence emission spectra for the control and the A240D and E241A mutants. **a** Low-temperature fluorescence emission spectra following excitation at 440 nm. **b** Low-temperature fluorescence emission spectra following excitation at 580 nm. In panels a and b, the black line represents control, the black dotted line shows A240D, and the blue line represents E241A. Spectra are the average of three independent experiments and have been normalized to the PS I peak (725 nm).


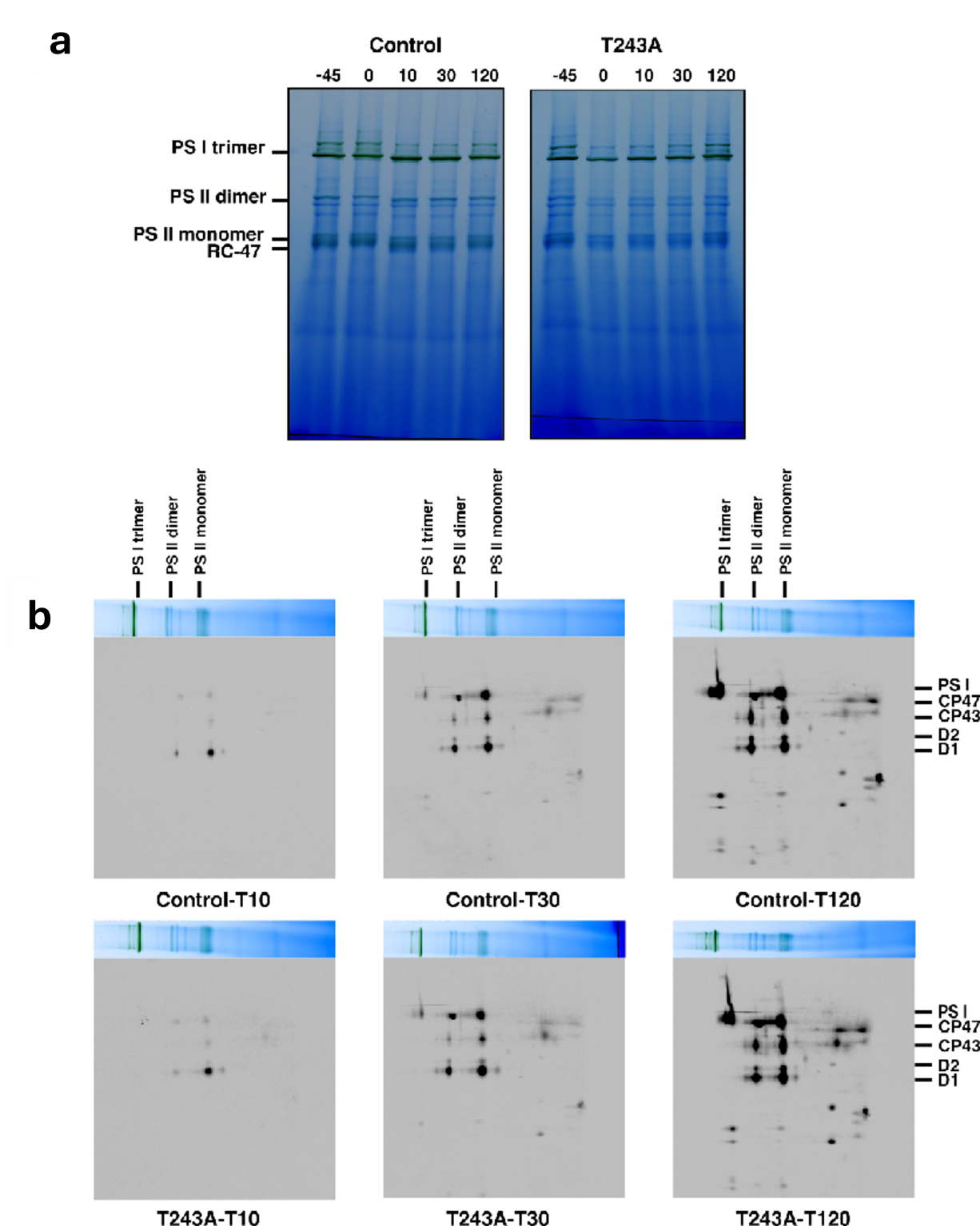


**Fig. S3** Analysis of photodamage and recovery in isolated thylakoid membranes. Control and T243A cells at 10 µg mL^–1^ chlorophyll *a* were subjected to high light (2000 µmol photons m^–2^ s^–1^) for 45 min, followed by recovery in the presence of ^35^S-Met under low light (30 µmol photons m^–2^ s^–1^) for 120 min. **a** After thylakoid extraction and solubilization, complexes were separated by BN-PAGE. Samples were taken before the onset of the 45 min high-light treatment (-45 min) and at the end of the high-light treatment (0 min); and also at 10 min, 30 min and 120 min during the low-light recovery period. The bands corresponding to PS I trimers, PS II dimers, PS II monomers and RC47 complexes are indicated. **b** Autoradiograph of BN-PAGE and SDS-PAGE two-dimensional analysis at 10 min (left panels), 30 min (middle panels) and 120 min (right panels) during the recovery period. The PS I, D1, D2, CP47 and CP43 bands are indicated. Note the PS I band is comprised of the PsaA and PsaB subunits.


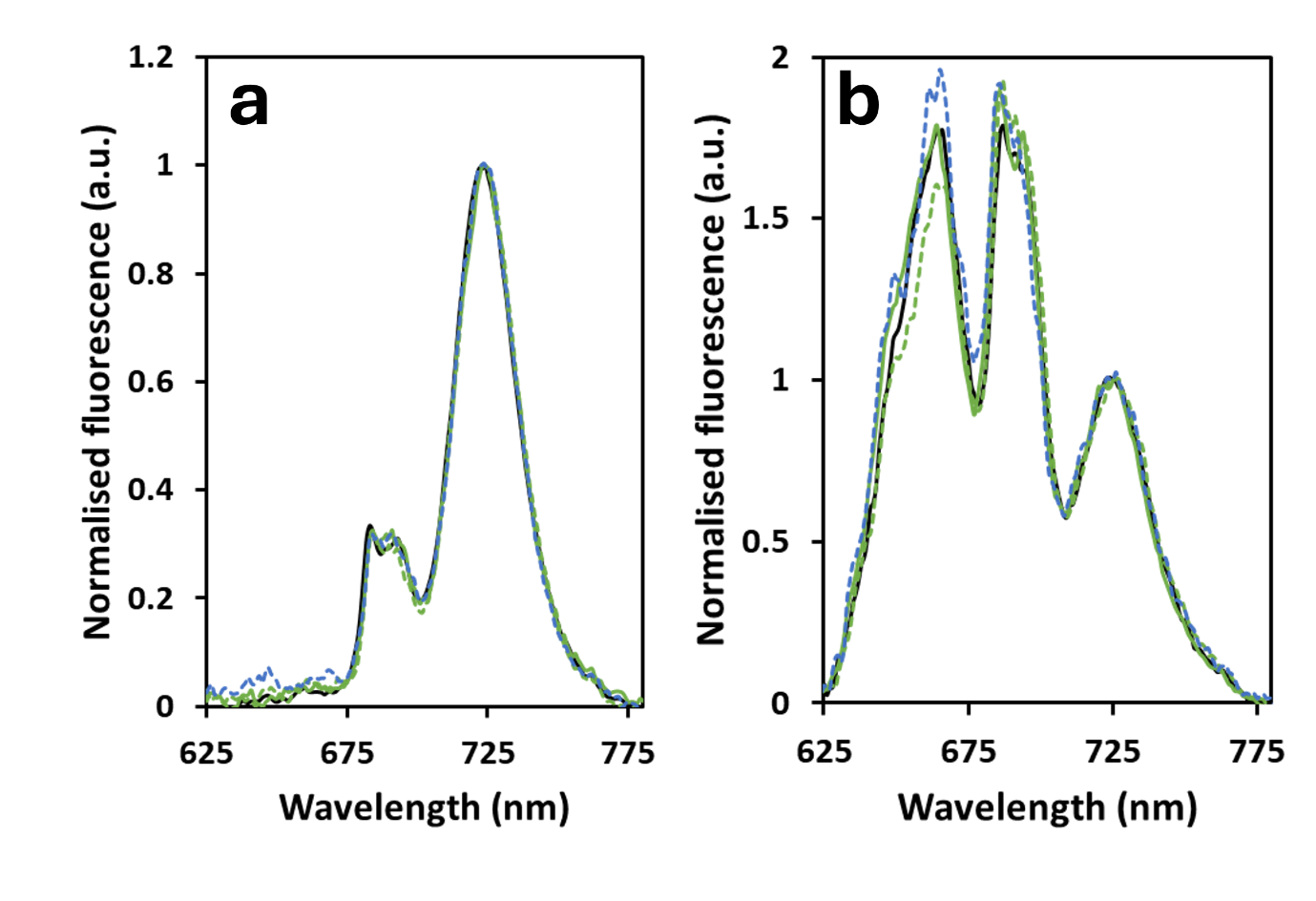


**Fig. S4** Low-temperature (77 K) fluorescence emission spectra for the control and the E242A, E242D and T243A mutants. **a** Low-temperature fluorescence emission spectra following excitation at 440 nm. **b** Low-temperature fluorescence emission spectra following excitation at 580 nm. In panels a and b, the black line represents control, the green line represents E242A, the green dashed line shows E242D, and the blue dashed line represents T243A. Spectra are the average of three independent experiments and have been normalized to the PS I peak (725 nm).

**
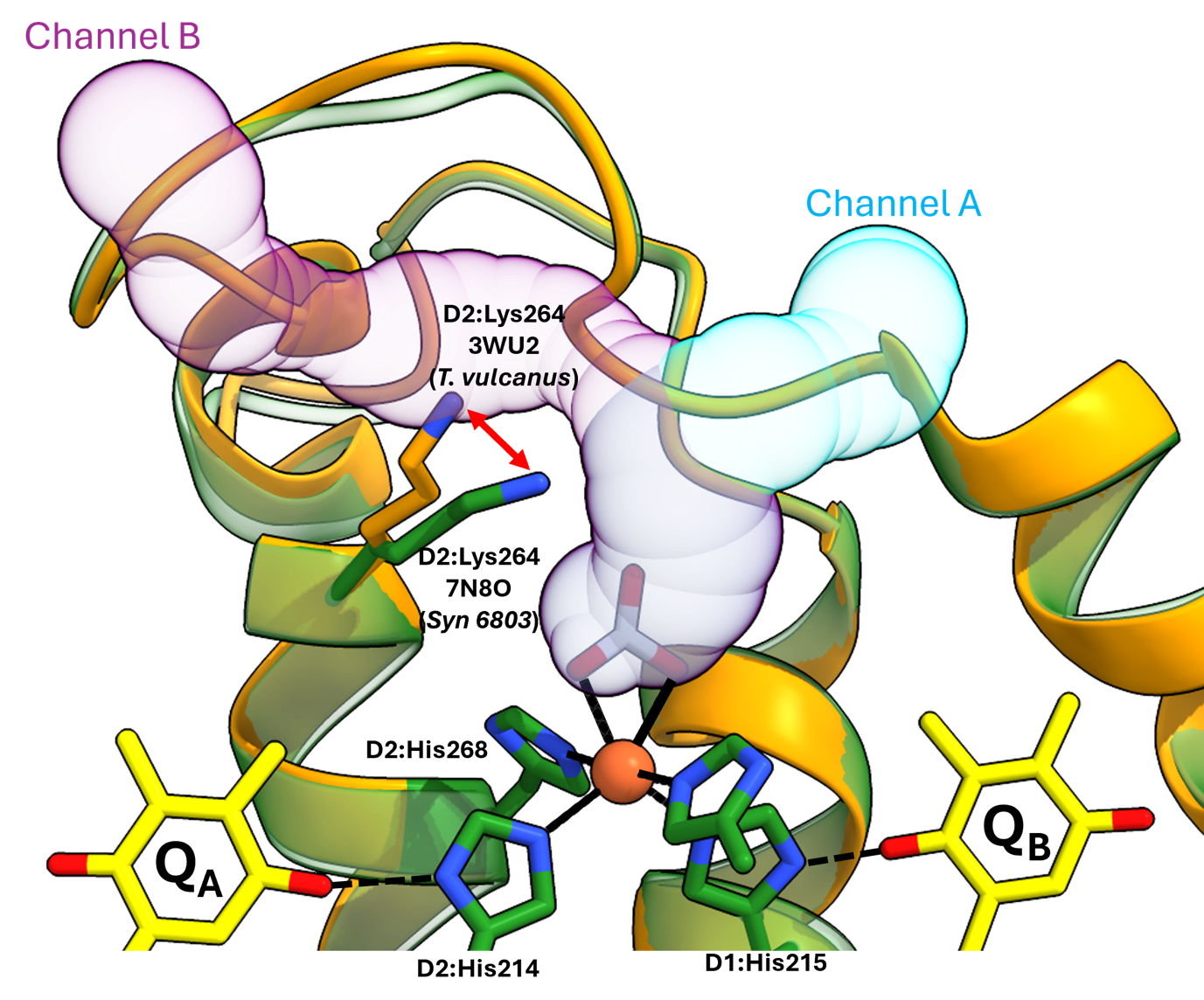
**

**Fig. S5** Overlay of the DE-Loop regions of the D2 protein in the vicinity of D2-Lys264 from *Thermostichus vulcanus* (PDB: 3WU2) and *Synechocystis* sp. PCC 6803 (PDB: 7N8O). The D2 sequence and position of D2-Lys264 from *T. vulcanus* is shown in orange. The D2 sequence and position of D2-Lys264 from *Synechocystis* 6803 is shown in green. The A and B channels identified by CAVER [see (Jurcik et al. 2018)] in Fig. 1 are shown with D2-Lys264 from *T. vulcanus* obstructing Channel B. The non-heme iron is shown in orange with the His ligands from D1 and D2 and the bicarbonate bidentate ligand also shown. Coordination bonds are shown as black lines and hydrogen bonds as dashed black lines. The primary and secondary quinone electron acceptors Q_A_ and Q_B_ are shown in yellow. Oxygens are shown in red and nitrogen atoms are shown in blue.

**
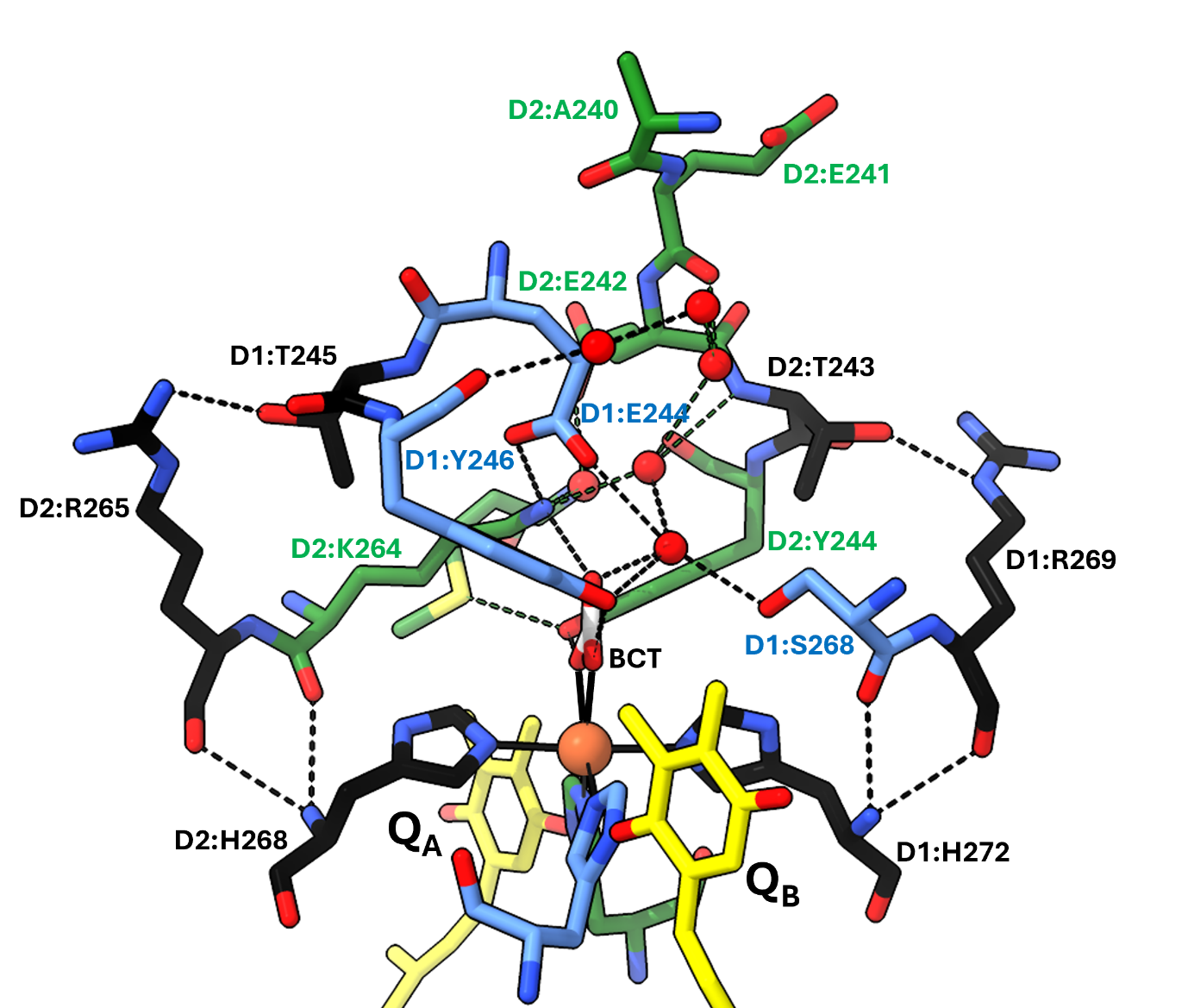
**

**Fig. S6** Location of D2-Arg265 and D1-Arg269 in proximity to the non-heme iron and bicarbonate-binding environment of the acceptor side of PS II. BCT represents bicarbonate, Fe is the non-heme iron and red spheres indicate water molecules. Q_A_ and Q_B_ are shown in yellow. Oxygen atoms are shown in red, nitrogen atoms are shown in blue. D1 residues are in blue and D2 residues are in green. Key residues in the Thr-Arg-His motif are shown in black. Dashed black lines indicate putative hydrogen bonds or salt bridge interactions. The coordinate bonds to the non-heme iron are shown in solid black lines.

| **Table S1** Primer sequences used in QuikChange site-directed mutagenesis of the D2 protein to make the A240D, E241A, E242A, E242D and T243A mutants | |
| --- | --- |
| Primer | Primer Sequence (5’ – 3’) |
| D2-A240D-US^a^ | GCATTTGAACCCACCCAAGATGAAGAAACCTATTCCATGG |
| D2-A240D-DS^b^ | CCATGGAATAGGTTTCTTCATCTTGGGTGGGTTCAAATGC |
| D2-E241A-US | TGAACCCACCCAAGCAGCAGAAACCTATTCCATGG |
| D2-E241A-DS | CCATGGAATAGGTTTCTGCTGCTTGGGTGGGTTCA |
| D2-E242A-US | TCACCATGGAATAGGTTGCTTCTGCTTGGGTGGG |
| D2-E242A-DS | CCCACCCAAGCAGAAGCAACCTATTCCATGGTGA |
| D2-E242D-US | ACCATGGAATAGGTGTCTTCTGCTTGGGTGGGTTC |
| D2-E242D-DS | GAACCCACCCAAGCAGAAGACACCTATTCCATGGT |
| D2-T243A-US | CACCATGGAATAGGCTTCTTCTGCTTGGGTGGGT |
| D2-T243A-DS | ACCCACCCAAGCAGAAGAAGCCTATTCCATGGTG |
| ^a^US: upstream  ^b^DS: downstream | |

| **Table S2** Kinetic analysis of the decay of chlorophyll *a* fluorescence following three saturating actinic flashes spaced at 200 ms intervals in the A240D, E241A, E242A, E242D and T243A mutants^a^ | | | | | | |
| --- | --- | --- | --- | --- | --- | --- |
| strain | fast component | | intermediate component | | slow component | |
|  | rate | amplitude (%) | rate | amplitude | rate | amplitude (%) |
|  | (t_1/2_ = µs) |  | (t_1/2_ = ms) | (%) | (t_1/2_ = s) |  |
| Control | 321 ± 24.0 | 64.0 ± 1.0 | 3.0 ± 0.0 | 27.0 ± 1.0 | 8.0 ± 2.0 | 9.0 ± 0.0 |
| A240D | 320 ± 31.0 | 67.0 ± 6.0 | 7.0 ± 0.4 | 24.3 ± 1.0 | 8.4 ± 3.0 | 8.5 ± 1.1 |
| E241A | 370 ± 18.2 | 71.0 ± 4.0 | 8.2 ± 0.6 | 20.4 ± 3.9 | 9.3 ± 2.0 | 8.0 ± 0.4 |
| E242A | 372 ± 16.0 | 56.0 ± 3.2 | 9.0 ± 0.8 | 33.0 ± 1.3 | 3.0 ± 1.1 | 11.0 ± 1.9 |
| E242D | 383 ± 41.0 | 54.0 ± 2.3 | 4.0 ± 0.3 | 36.0 ± 2.4 | 11.0 ± 1.4 | 9.0 ± 0.1 |
| T243A | 475 ± 19 | 53.0 ± 0.3 | 5.0 ± 0.2 | 28.0 ± 1.1 | 7.0 ± 1.6 | 19.0 ± 0.8 |
| ^a^Kinetic analyses were performed according to (Vass et al. 1999). Data are the average of three independent experiments, and the standard error of the mean for the calculated rates and amplitudes is shown. | | | | | | |

| **Table S3** Kinetic analysis of the decay of chlorophyll *a* fluorescence after a single turnover flash for cells treated with 25 mM formate, 15 mM bicarbonate, or 15 mM bicarbonate and 25 mM formate in the A240D, E241A, E242A, E242D and T243A mutants^a^ | | | | | | | |
| --- | --- | --- | --- | --- | --- | --- | --- |
| treatment | strain | fast component | | intermediate component | | slow component | |
|  |  | rate | amplitude | rate | amplitude | rate | amplitude |
|  |  | (t_1/2_ = µs) | (%) | (t_1/2_ = ms) | (%) | (t_1/2_ = s) | (%) |
| formate | Control | 324 ± 16 | 62.0 ± 0.0 | 7.5 ± 2.0 | 26.0 ± 0.3 | 3.1 ± 0.5 | 13.0 ± 0.8 |
|  | A240D | 277 ± 27 | 63.0 ± 1.0 | 6.7 ± 0.2 | 28.0 ± 0.1 | 6.2 ± 1.0 | 8.0 ± 0.5 |
|  | E241A | 677 ± 55 | 46.0 ± 3.0 | 11.4 ± 0.2 | 27.0 ± 0.0 | 0.8 ± 1.0 | 26.0 ± 2.0 |
|  | E242A | 341 ± 47 | 57.0 ± 0.2 | 6.0 ± 0.2 | 35.0 ± 0.1 | 6.0 ± 1.0 | 8.0 ± 0.3 |
|  | E242D | 457 ± 57 | 64.0 ± 2.4 | 11.0 ± 1.4 | 27.0 ± 1.4 | 4.3 ± 0.9 | 10.0 ± 1.0 |
|  | T243A | 1006 ± 74 | 45.0 ± 0.9 | 13.0 ± 2.8 | 39.0 ± 0.8 | 3.0 ± 1.2 | 16.0 ± 0.2 |
| HCO_3_^-^ | Control | 261 ± 10 | 62.0 ± 1.6 | 3.6 ± 1.2 | 27.0 ± 3.5 | 6.2 ± 0.0 | 11.0 ± 1.9 |
|  | A240D | 203 ± 15 | 60.0 ± 1.0 | 2.6 ± 0.0 | 26.0 ± 2.0 | 5.5 ± 1.2 | 9.0 ± 1.3 |
|  | E241A | 333 ± 38 | 60.0 ± 1.0 | 3.0 ± 0.3 | 28.0 ± 1.0 | 3.6 ± 1.0 | 10.0 ± 3.0 |
|  | E242A | 299 ± 29 | 61.0 ± 0.5 | 6.0 ± 0.4 | 31.0 ± 0.3 | 5.0 ± 0.2 | 8.0 ± 0.2 |
|  | E242D | 312 ± 31 | 55.0 ± 2.7 | 3.0 ± 0.6 | 37.0 ± 4.1 | 7.0 ± 2.3 | 8.0 ± 1.5 |
|  | T243A | 396 ± 43 | 37.0 ± 5.7 | 3.0 ± 0.3 | 50.0 ± 8.0 | 4.0 ± 0.8 | 14.0 ± 2.3 |
| formate + HCO_3_^-^ | Control | 250 ± 34 | 59.0 ± 2.5 | 3.2 ± 0.3 | 29.0 ± 0.4 | 3.9 ± 1.0 | 12.0 ± 2.9 |
|  | A240D | 259 ± 61 | 64.0 ± 1.0 | 3.0 ± 0.0 | 27.0 ± 1.0 | 6 ± 0.0 | 9.0 ± 0.0 |
|  | E241A | 384 ± 78 | 63.0 ± 2.0 | 3.0 ± 0.0 | 31.0 ± 2.0 | 5.6 ± 1.0 | 9.0 ± 0.0 |
|  | E242A | 305 ± 17 | 59.0 ± 0.3 | 6.0 ± 0.3 | 33.0 ± 0.6 | 4.0 ± 1.0 | 8.0 ± 0.5 |
|  | E242D | 404 ± 12 | 53.0 ± 0.7 | 3.0 ± 1.0 | 40.0 ± 0.1 | 5.0 ± 0.9 | 7.0 ± 0.7 |
|  | T243A | 416 ± 11 | 40.0 ± 3.4 | 3.0 ± 0.1 | 48.0 ± 4.0 | 6.0 ± 3.0 | 12.0 ± 0.7 |
| ^a^Kinetic analyses were performed according to (Vass et al. 1999). Data are the average of three independent experiments and the standard error of the mean for the calculated rates and amplitudes is shown. | | | | | | | |

**References**

Jurcik A, Bednar D, Byska J, Marques SM, Furmanova K, Daniel L, Kokkonen P, Brezovsky J, Strnad O, Stourac J, Pavelka A, Manak M, Damborsky J, Kozlikova B (2018) CAVER Analyst 2.0: Analysis and visualization of channels and tunnels in protein structures and molecular dynamics trajectories. Bioinformatics 34:3586–3588. https://doi.org/10.1093/bioinformatics/bty386

Vass I, Kirilovsky D, Etienne A-L (1999) UV-B radiation-induced donor- and acceptor-side modifications of Photosystem II in the cyanobacterium *Synechocystis* sp. PCC 6803. Biochemistry 38:12786–12794. https://doi.org/10.1021/bi991094w
